# A Digital Diabetes Self-Management Education and Support Program Integrated With Continuous Glucose Monitoring for Type 2 Diabetes: Randomized Controlled Trial

**DOI:** 10.2196/78321

**Published:** 2026-05-14

**Authors:** Ashley Berthoumieux, Jeanean B Naqvi, Sean Zion, Jenna Napoleone, Amanda McGuill, Christian J Cerrada, Hyun Jung Lee, Timothy C Dunn, David Kerr, Carolyn Bradner Jasik, Sarah Linke

**Affiliations:** 1 Omada Health, 500 Sansome St, #200, San Francisco, CA, 94111, United States, 1 4128746505; 2 Evidation Health, Inc., San Mateo, CA, United States; 3 Abbott Diabetes Care, Inc., Alameda, CA, United States; 4 Sutter Health Center for Health Systems Research, Santa Barbara, CA, United States

**Keywords:** diabetes self-management education and support, diabetes education, digital health, continuous glucose monitoring, time in range, chronic disease management

## Abstract

**Background:**

Previous research has demonstrated that the use of continuous glucose monitoring (CGM) can improve glycemic control in people with type 2 diabetes when used regularly alongside a digital diabetes self-management education and support (DSMES) program. However, to date, there is limited evidence showing the benefits of a digitally delivered DSMES program combined with real-time CGM for adults with type 2 diabetes.

**Objective:**

The objective of this study is to evaluate the impact of a DSMES program coupled with CGM on hemoglobin A_1c_ (HbA_1c_) and CGM-derived glycemic measures compared to usual care for adults with type 2 diabetes over 6 months.

**Methods:**

Participants with type 2 diabetes and HbA_1c_ of 8% or higher (64 mmol/mol) who were not using mealtime bolus insulin (aged 26‐83 y; mean HbA_1c_ 9.6%, SD 1.4% [mean 81.2 mmol/mol, SD 15.8 mmol/mol]) were randomly assigned to a digital DSMES+CGM integrated solution (n=51) or usual care (n=49) for 6 months. The primary outcome was HbA_1c_. The secondary outcomes were CGM-derived glycemic measures, including glucose management indicator, percent time in range 70 to 180 mg/dL, percent time above range (>180 mg/dL), percent time below range (<70 mg/dL), and mean glucose. Linear mixed effects models were used for intention-to-treat analyses.

**Results:**

HbA_1c_ was lower among the intervention group versus the usual care group at 3 months (difference=−0.7%, 95% CI −1.4% to −0.1% or difference=−8.1 mmol/mol, 95% CI −15.5 to −0.7 mmol/mol; *P*=.03) and at 6 months (difference=−0.6%, 95% CI −1.4% to 0.2% or difference=−6.9 mmol/mol, 95% CI −15.7 to 1.9 mmol/mol; *P*=.12) but only reached statistical significance at 3 months. CGM-derived glycemic measures, including glucose management indicator (difference=−0.9%, 95% CI −1.7% to −0.1%; *P*=.03), time in range (difference=14.6%, 95% CI 1.0% to 28.2%; *P*=.04), time above range (difference=−14.9%, 95% CI −29.0% to −0.9%; *P*=.04), and mean glucose (difference=−36.4 mg/dL, 95% CI −70.0 to −2.9 mg/dL; *P*=.03), also significantly improved for the intervention group versus the usual care group at 6 months.

**Conclusions:**

The combination of digital DSMES+CGM is effective for supporting adults with type 2 diabetes in managing their condition and has the potential to reduce the risk of long-term health complications.

## Introduction

Type 2 diabetes is a chronic disease that affects the body’s ability to regulate and use insulin effectively, leading to high blood glucose levels. Persistent hyperglycemia, as estimated by hemoglobin A_1c_ (HbA_1c_) [1], increases the risk of severe diabetes complications, including but not limited to vision loss, renal failure, and cardiovascular disease [2]. As the incidence and prevalence of type 2 diabetes continue to rise nationwide, impacting more than 34 million adults in 2021 [3], there is a pressing need to develop and scale accessible, evidence-based diabetes management programs to alleviate the significant societal and individual burden of the chronic condition.

Diabetes self-management education and support (DSMES) is an effective approach for helping individuals practice diabetes self-care with intensive counseling and support [4,5]. DSMES programs are scalable using technology-enabled platforms [6], allowing people with chronic diseases to access DSMES programs from their personal mobile devices (eg, smartphones, tablets, laptops) and eliminate the need to travel to physical facilities for diabetes education sessions. This convenience potentially increases access and availability, thereby reaching more participants than traditional in-person programs [7].

The introduction of continuous glucose monitoring (CGM) devices has revolutionized diabetes management by providing real-time glucose readings without painful fingersticks. For those taking insulin or at high risk for complications, CGMs help monitor fluctuations in blood glucose, informing potential changes to their diet or medication regimens [8]. Studies have demonstrated that CGM can be a cost-effective option compared to conventional self-monitoring of blood glucose [9-11] and can improve glycemic control in people with type 2 diabetes when used regularly alongside DSMES programs [12].

However, previous research studies evaluating DSMES programs integrated with CGM have predominantly focused on in-person programs and short-term intermittent use of CGM for less than 8 weeks [13-16]. A few observational studies have explored the effects of a telemedicine lifestyle program for diabetes that included a CGM offering [17,18], but CGM use among participants was short term and the findings could not be extrapolated to show a cause-effect relationship between the virtually integrated approach and improved glycemic control.

Nevertheless, a meta-analysis of randomized controlled trials (RCTs) evaluating the clinical effectiveness of telemedicine for diabetes revealed that virtual health care services are generally more effective for diabetes care management than in-person care alone [19]. People participating in digital diabetes care programs have seen improved clinical outcomes (eg, HbA_1c_ reduction) and reduced medical expenditures [20-22], but clinical trials evaluating many of these digital programs have not reported CGM data related to their interventions for type 2 diabetes [23-25].

Despite growing observational research demonstrating the effectiveness of digital DSMES and CGM, to date, no RCTs have shown the benefits of a well-integrated solution that combines a digitally delivered DSMES program with longer-term, real-time CGM for adults with type 2 diabetes. Therefore, the aim of this RCT was to evaluate the impact of the integrated solution—defined as a digital DSMES program+CGM—on HbA_1c_, CGM-derived glycemic measures, and other relevant diabetes management outcomes compared to usual care over 6 months.

## Methods

### Study Design

This single-blind, parallel, decentralized RCT was designed to assess the efficacy of a digital DSMES+CGM integrated solution on glycemic management among adults with type 2 diabetes not on mealtime bolus insulin therapy, in accordance with the intended labeling of the FreeStyle Libre (FSL) 14-Day CGM system (Abbott Diabetes Care, Inc). The primary objective of this study was to evaluate the relative improvement in HbA_1c_ from baseline to 3 and 6 months of the digital DSMES+CGM intervention condition compared with a usual care control condition.

### Ethical Considerations

The protocol was approved by the WCG Institutional Review Board (IRB Tracking ID: 20220021) and registered on ClinicalTrials.gov (NCT05368454). All participants provided electronic informed consent. All research data were deidentified and coded with unique identifiers, and the data were transmitted using secure encrypted protocols and stored on encrypted disks on secure servers. Participants received up to a total of US $250 for completing all study activities.

### Participants

To meet the inclusion criteria, participants had to be at least 18 years old, living in the United States, have English as their primary language, have a compatible smartphone for access to the digital DSMES program, and have a self-reported type 2 diabetes diagnosis for at least 6 months prior to enrollment. Additionally, they needed a laboratory-confirmed HbA_1c_ level of 8% or higher (64 mmol/mol) at baseline, a treatment regimen that included a combination of basal insulin, oral medications, and diet and exercise, and a report being compliant with their diabetes management plan. If on a medication regimen, they must have been on their current medication regimen for at least 3 months prior to enrollment and have been willing to adhere to their regimen throughout the study. During the initial recruitment period (June-October 2022), participants were also required to have a percent time in range (TIR; 70‐180 mg/dL) of <60% prior to enrollment, which was eventually removed from the eligibility criteria to reduce participant burden and improve study activity workflow during the remainder of the recruitment period (October 2022-May 2023).

Exclusion criteria included type 1 diabetes or a history of diabetic ketoacidosis, cardiovascular issues within 3 months before enrollment (ie, transient ischemic attack or stroke, heart attack, hospitalization for congestive heart failure), recent (within 6 mo before enrollment) or planned cancer treatment, a self-reported condition leading to a life expectancy <12 months, renal impairment, alcohol or substance abuse issue or dependency, bariatric surgery or organ transplant within 6 months before enrollment, and pregnancy or intentions of conceiving during the study period (female participants). Other exclusion criteria included mealtime bolus insulin therapy, premixed insulin formulations or a continuous subcutaneous infusion of insulin, routine regimen of glucocorticoids or psychotropic medications, skin conditions that could interfere with CGM device placement, diagnostic imaging (ie, X-ray, magnetic resonance imaging, computed tomography) scheduled while wearing a CGM for measurement purposes, and visual impairment that could interfere with viewing of CGM data or use of the digital DSMES program.

### Recruitment

We recruited individuals to join this decentralized trial through Evidation (Evidation Health, Inc.), an online health platform through which users connect their digital health tools, such as fitness apps and wearable activity trackers, in exchange for monetary incentives. Targeted emails and platform posts were sent to existing Evidation members who had agreed to receive communications about studies that may be relevant to them based on the health and lifestyle information they had provided to the platform. We also recruited non-Evidation members, targeting individuals in geographic areas with the highest prevalence of type 2 diabetes through paid advertisements on Google Search and Facebook and forum posts on Reddit between February and May 2023.

### Eligibility Screening

Those who passed an initial screening proceeded to sign an electronic informed consent form for the study and completed an online baseline survey, which collected their demographic information, health and diabetes history, and patient-reported outcomes. A secondary screening visit was performed to confirm that potential participants met the additional eligibility criteria of an HbA_1c_ level of 8% or higher (64 mmol/mol) and, prior to October 2022, a TIR (70‐180 mg/dL) of <60%. Contracted vendors (PCM Trials/Act for Health, Inc, and Hawthorne Effect, Inc) deployed certified and trained clinicians to conduct home visits in cities and rural areas across the United States, eliminating the need for participants to travel to a physical site or clinic for clinical assessments. During the home visits, they collected up to 4 mL of blood using venipuncture, anthropometric measurements, resting blood pressure, and a list of participants’ cardiometabolic medications. They also applied and activated 2 FSL Pro CGM sensors on the participants for measurement purposes, and participants were instructed to wear the FSL Pro CGM sensors for up to 14 days while blinded to the data. Afterwards, participants mailed the devices to the study team for data download and TIR analysis. The contracted clinicians mailed the blood specimens to Quest Diagnostics laboratory for HbA_1c_ analysis. Participants who had a laboratory-confirmed HbA_1c_ of 8% or higher (64 mmol/mol) and a TIR (70‐180 mg/dL) of <60% if recruited prior to mid-October 2022 were randomized and considered enrolled in the study.

### Randomization and Masking

Each eligible participant was randomized to 1 of the 2 conditions (digital DSMES+CGM or usual care) according to an Evidation-controlled algorithm that used randomly permuted blocks of fixed size. Randomization was stratified by gender to ensure nearly equal sample sizes for the 2 treatment arms for each gender.

The assessment staff members (ie, clinicians conducting home visits, Quest Diagnostics) and the Evidation-designated statistician that developed the statistical analysis plan were blinded to the intervention assignment during the study period. Adverse-event adjudicators, designated research personnel at Evidation and Omada, and Omada coaching staff were not blinded. Participants were not blinded to their assignment but were blinded to their HbA_1c_ levels and CGM blood glucose values collected by FSL Pro sensors.

### Digital DSMES+CGM Integrated Solution

Participants randomized to the digital DSMES+CGM intervention arm completed an application for the Omada for Diabetes program, a digitally delivered DSMES program that has achieved accreditation from the National Committee for Quality Assurance’s Population Health Program and Accreditation and the Association of Diabetes Care and Education Specialists. The program pairs asynchronous health coaching from a care team comprising certified Diabetes Prevention Program Lifestyle Coaches and Certified Diabetes Care and Education Specialists (CDCESs) with a virtual platform accessed through a website or mobile app available on web-enabled devices. Upon enrollment, members receive cellularly connected Food and Drug Administration–approved devices, such as body weight scales and CGMs, along with tailored diabetes self-management and lifestyle modification support under the guidance of their care team. The care team interacts with members asynchronously through messaging on the virtual platform, regularly monitoring participants’ engagement and biometric data collected during the program and using it to provide tailored feedback on diabetes self-management through proactive outreach and responding to member questions. CGM data are continuously collected in real time, and when scanned, glucose data are sent to the program app, where they are available for members to see patterns, track progress, and get personalized recommendations from their assigned coach. Additional program features include weekly lessons with nutrition and lifestyle content, support around personalized goal setting, and online peer communities. The weekly curriculum lessons and all program features are accessible on both computers and mobile devices, enabling members to engage at their preferred times, frequency, and with the tools and resources they find most useful [26].

After submitting an application to join the digital DSMES program as research members, digital DSMES+CGM participants completed their account setup through a one-time consultation with a board-certified physician from CirrusMD, a contracted third-party telehealth provider that independently determined whether a CGM prescription was clinically appropriate for an individual. CirrusMD physicians evaluated members and prescribed 14 Abbott FSL 14-Day CGM sensors as part of the integrated solution, which were sent directly to members’ homes to cover 6 months of continuous wear while participating in the digital DSMES program. During the follow-up assessment periods (3-mo and 6-mo timepoints), participants in the intervention condition were instructed to wear their FSL Pro sensors and FSL 14-Day sensor at the same time. Unlike the FSL Pro sensors used for measurement of CGM-derived glycemic outcomes, members and their care team at Omada had full access to the CGM data collected through the FSL 14-Day sensors for 6 months.

### Usual Care Control Condition

Participants randomized to usual care were advised during the informed consent process to continue the diabetes treatment plan as recommended by their health care provider from screening to the end of the study period. Usual care included oral medications, basal insulin, or nonpharmacologic therapies such as diet and exercise, in addition to ongoing medical monitoring by their health care provider. During the follow-up assessment periods (3-mo and 6-mo timepoints), participants in the usual care control condition only needed to wear their FSL Pro sensors and did not receive FSL 14-Day sensors.

### Follow-Up Visits

Interim and poststudy follow-up visits occurred 3 and 6 months (±14 d) after randomization for all study participants, respectively. During these follow-up visits at participants’ homes, contracted clinicians performed blood draws, took anthropometric measurements, assessed resting blood pressure, and reviewed cardiometabolic medication regimens. At each follow-up visit, clinicians applied and activated 2 FSL Pro sensors on participants for measurement and data quality purposes. Study participants were then instructed to wear the sensors for up to 14 consecutive days while blinded to the data and to mail them back to the study team using a prepaid shipping envelope.

Return follow-up visits occurred outside of the ±14-day range if a repeat blood draw was needed. This transpired if there was no usable data from the sample or FSL Pro sensors through no fault of the participant (eg, mail delays causing the blood sample to be outside the testing window, sensor malfunction). Participants received compensation for all completed study visits.

### Measures

Study outcomes were compared between the digital DSMES program+CGM versus usual care at 3 and 6 months. The primary outcome was HbA_1c_ level. The secondary outcomes measured by the FSL Pro sensors included CGM 14-day % TIR, time above range (TAR; >180 mg/dL and >250 mg/dL), and time below range (TBR; <70 mg/dL). Furthermore, differences in mean blood glucose, SD of blood glucose, coefficient of variation, and glucose management indicator (GMI) were examined. Lastly, body weight, resting systolic and diastolic blood pressure, and diabetes distress [27] were examined. Engagement in the digital DSMES+CGM integrated solution was measured using 5 metrics: average number of meals tracked per week using the digital DSMES app, average number of physical activity bouts tracked per week (manually self-reported in the digital DSMES app or synced from a wearable device), average number of weigh-ins per week using the provided cellularly connected body weight scale, average number of messages sent to their care team per week, and median number of days in which participants had at least 1 CGM reading from the FSL 14-Day device (connected through the digital DSMES app).

### Statistical Analysis

Power was calculated assuming a −0.5% (5 mmol/mol) mean difference in HbA_1c_ between the digital DSMES+CGM condition and usual care from baseline to 6 months. Using an estimated SD of 1.0, a 2-tailed type I error rate of 0.05, and 80% power, the sample size required for the primary analysis was 126 participants in total (63 per group) [28]. Assuming an attrition rate of 20%, the adjusted sample size was 160 participants (80 per group). Therefore, since we reduced our recruitment goal during the active study period due to challenges with recruitment, we were underpowered to detect a statistically significant difference with our estimated effect size of 0.5%.

All analyses were conducted with R (version 4.2.3) [29]. Demographics and baseline clinical indicators (ie, age, BMI, sex, cardiovascular medication use, and type 2 diabetes medication use) were examined as potential covariates by exploring condition differences in each covariate and examining associations between each covariate and outcome. Given that there were no condition differences on any of the potential covariates, no covariates were included in the analyses.

Linear mixed effects models were used to analyze both the primary and secondary outcomes using intention-to-treat (ITT) methodology, given that these models utilize all available data [30]. The Little missing completely at random (MCAR) test [31] was nonsignificant for all primary and secondary outcomes, supporting the MCAR assumption for this analysis and indicating that imputation was not necessary. For the primary outcome model, fixed effects included timepoint, condition, and a timepoint-by-condition interaction. A random slope for time and a random intercept for participant were included. For the secondary outcome models, fixed effects included timepoint, condition, and a timepoint-by-condition interaction. Given that several of the models would not converge when a random slope for time was included, only a random intercept for participant was included in the models [32]. All models utilized restricted maximum likelihood estimation with an unstructured covariance matrix. Estimated marginal means were obtained from the models, and planned contrasts were conducted to examine differences by condition at 3 and 6 months.

In addition to the main analysis, we conducted sensitivity analyses for all primary and secondary outcomes where we excluded participants (n=9) who were randomized to the intervention condition but never enrolled in the digital DSMES+CGM integrated solution (sample size n=42). These models utilized the same fixed and random effects for both the primary and secondary outcomes. Finally, engagement descriptives (mean and SD) were assessed among this group of intervention participants that enrolled in the digital solution (n=42).

## Results

### Baseline Characteristics

**Figure 1.**
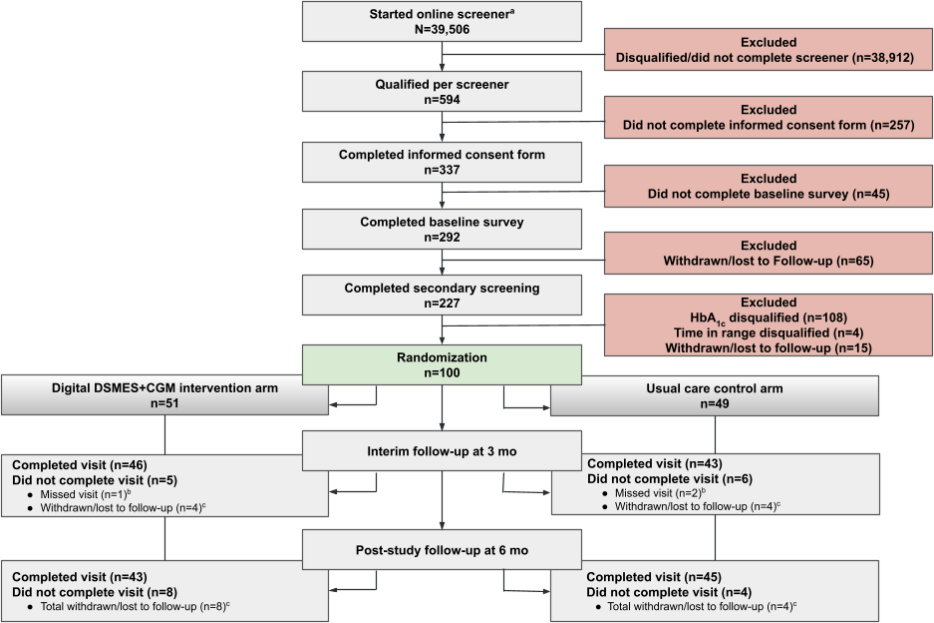
CONSORT (Consolidated Standards of Reporting Trials) flow diagram. ^a^Participants were recruited from Evidation Health’s online platform if they had previously agreed to receive communications about research studies when they signed up to become an Evidation member; the number reflected in this box is the number of participants who started filling out the screener survey to assess their potential eligibility for this decentralized RCT. ^b^Home visit was not done, but the research staff was able to maintain contact with participants who missed a 3-month assessment. ^c^Participants were either withdrawn due to ineligibility criteria or were marked lost to follow-up if the research staff was unable to reach participants after multiple attempts. CGM: continuous glucose monitoring; DSMES: diabetes self-management education and support; HbA_1c_: hemoglobin A_1c_; RCT: randomized controlled trial.

A total of 39,506 individuals were initially screened for eligibility (Figure 1 and Checklist 1). Of these, 594 were screened as eligible, and 292 individuals completed an informed consent form and the baseline survey. After participating in a secondary screening (n=227), 112 individuals were disqualified due to HbA_1c_ or TIR eligibility criteria, and 15 individuals were withdrawn or lost to follow-up. Thus, the final sample included 100 participants randomized to the digital DSMES+CGM intervention condition (n=51) or usual care (n=49). At 3 months, 90.2% (46/51) digital DSMES+CGM participants and 87.8% (43/49) usual care participants completed their follow-up. At 6 months, 84.3% (43/51) digital DSMES+CGM participants and 91.8% (45/49) usual care participants completed their follow-up. The level of missingness for the primary outcome (HbA_1c_) was 24% at 3 months and 15% at 6 months, and the level of missingness for the secondary outcomes ranged from 12% to 14% at 3 months and from 13% to 23% at 6 months.

Baseline demographic and clinical characteristics of the study population are presented in Table 1. Across conditions, participants were 50.7 (SD 10.8) years of age on average, and the average weight was 215.9 (SD 45.1) pounds. Most participants were White (55/100, 55%) and female (70/100, 70%), and half had less than a 4-year college degree (50/100, 50%). The median household income was between US $50,000 and $74,999. Mean baseline HbA_1c_ was 9.6% (SD 1.4%) or 81.2 (SD 15.8) mmol/mol, and % TIR (70‐180 mg/dL) was 33.6 (SD 28.0). Of members who reported taking diabetes medication at one or more of the 3 study visits (n=95), 86 (91%) reported a change in their medication over time. Specifically, a total of 53% (50/95) of members started a new medication and 58% (55/95) of members stopped a medication during the study period, with 35% (33/95) of members increasing the total number of medications reported and 42% (40/95) decreasing the total number of medications reported during the study period. There were no significant differences in baseline characteristics (including baseline diabetes medications) or in the number of members who started or stopped a medication, used insulin, increased or decreased the number of medications reported, or had any medication change during the study period between the 2 conditions. When looking only at members who took oral medication only at baseline (n=67), there were no differences in baseline characteristics between the 2 conditions.

**Table 1.** Baseline characteristics of study participants by condition.

Characteristic	Digital DSMES^a^+CGM^b^ (n=51)	Usual care (n=49)	Overall (N=100)
Age (y), mean (SD)	51.7 (10.2)	49.5 (11.5)	50.7 (10.8)
Sex (female), n (%)	35 (68.6)	35 (71.4)	70 (70)
Race or ethnicity, n (%)			
White	29 (56.9)	26 (53.1)	55 (55)
Black/African American	11 (21.6)	9 (18.4)	20 (20)
Hispanic/Latino/Spanish	4 (7.8)	8 (16.3)	12 (12)
Another race	7 (13.7)	6 (12.2)	13 (13)
Education (<4-y college degree), n (%)	28 (54.9)	22 (44.9)	50 (50)
Household income, median	US $50,000-$74,999	US $50,000-$74,999	US $50,000-$74,999
Duration of type 2 diabetes (y), mean (SD)	10.4 (7.5)	10.5 (7.6)	10.5 (7.5)
Diabetes medication, n (%)			
GLP-1 injection	13 (25.5)	8 (16.3)	21 (21)
Oral medication only	31 (60.8)	36 (73.5)	67 (67)
Diet and exercise only	6 (11.8)	2 (4.1)	8 (8)
Basal insulin and oral medication	1 (2)	3 (6.1)	4 (4)
Weight (lb), mean (SD)	213.1 (40.5)	218.6 (49.6)	215.9 (45.1)
BMI (kg/m^2^), mean (SD)	34.2 (5.8)	35.9 (7.9)	35.0 (6.9)
HbA_1c_^c^ (%), mean (SD)	9.5 (1.4)	9.7 (1.5)	9.6 (1.4)
HbA_1c_ (mmol/mol), mean (SD)	80.1 (15.5)	82.2 (16.2)	81.2 (15.8)
% TIR^d^ (70‐180 mg/dL), mean (SD)	36.0 (28.5)	31.0 (27.5)	33.6 (28.0)
% TAR^e^ (>180 mg/dL), mean (SD)	63.0 (30.1)	68.3 (28.4)	65.5 (29.3)
% TBR^f^ (<70 mg/dL), mean (SD)	1.0 (3.4)	0.8 (2.5)	0.9 (3.0)
Mean glucose (mg/dL), mean (SD)	219.1 (68.9)	236.1 (75.8)	227.3 (72.4)
GMI^g^ (%), mean (SD)	8.6 (1.6)	9.0 (1.8)	8.8 (1.7)

a DSMES: diabetes self-management education and support.

b CGM: continuous glucose monitoring.

c HbA_1c_: hemoglobin A_1c_.

d TIR: time in range.

e TAR: time above range.

f TBR: time below range.

g GMI: glucose management indicator.

### Engagement Metrics (Digital DSMES+CGM Group Only)

Of those who enrolled in the digital DSMES+CGM integrated solution (n=42), the mean number of meals tracked was 4.6 (SD 6.7) per week, and the mean number of physical activity bouts recorded was 4.3 (SD 3.1) per week within the digital DSMES app. Participants also weighed themselves an average of 3.3 (SD 2.5) times per week using the cellularly connected body weight scale as part of the digital DSMES program. Participants sent an average of 1.2 (SD 1.1) messages to their care team per week. The median number of days per week in which participants had at least 1 CGM reading from the FSL 14-Day sensor was 4.8.

### Primary Outcome: HbA_1c_

**Figure 2.**
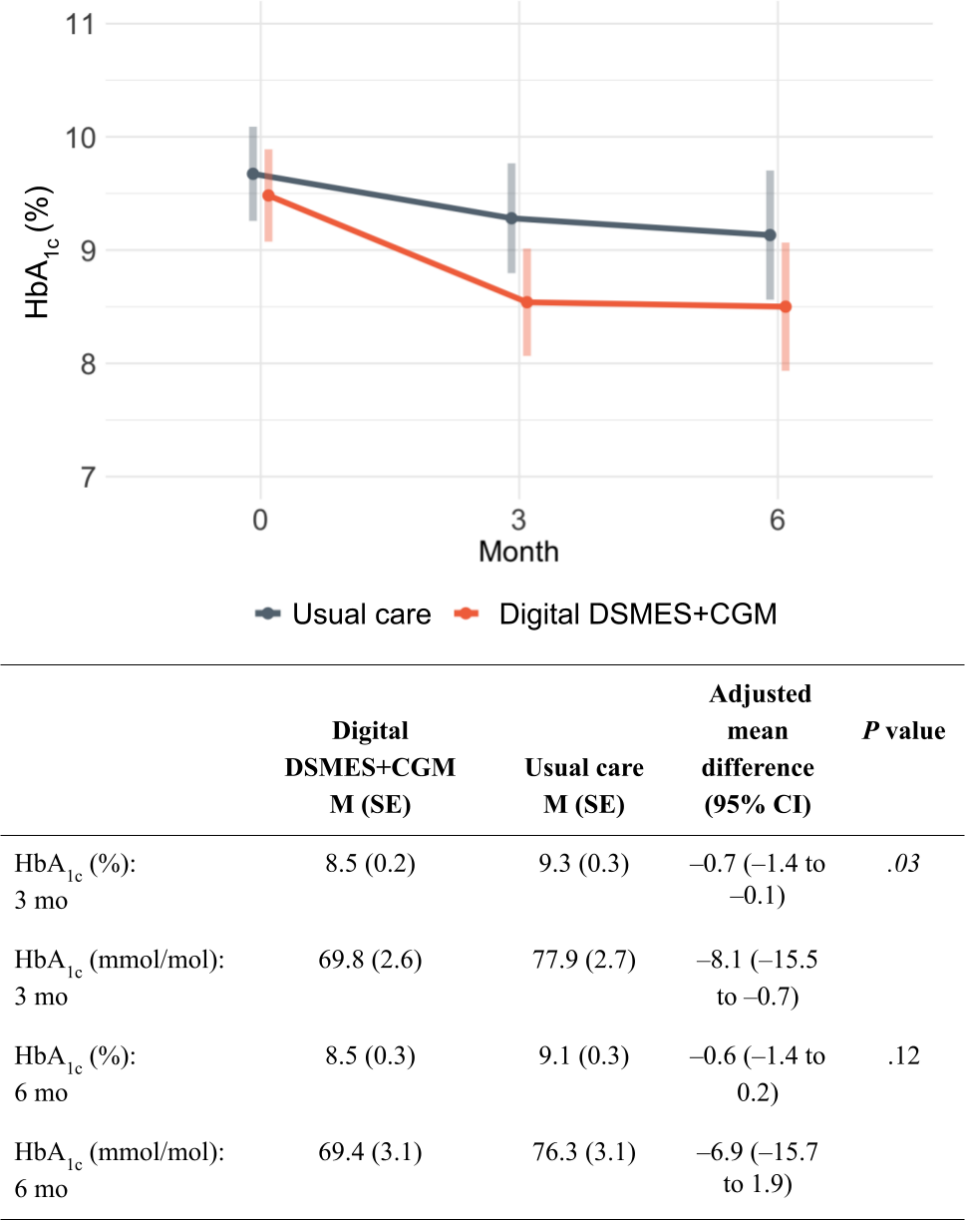
Change in mean hemoglobin A_1c_ over time by condition. *P* values <.05 are italicized. CGM: continuous glucose monitoring; DSMES: diabetes self-management education and support; HbA_1c_: hemoglobin A_1c_; M: mean.

The results for HbA_1c_ are presented in Figure 2. Participants in the digital DSMES+CGM condition had significantly lower HbA_1c_ than those in usual care at 3 months (−0.7%, 95% CI −1.4% to −0.1% or −8.1 mmol/mol, 95% CI −15.5 to −0.7 mmol/mol; *P*=.03) and lower, nonsignificant reductions in HbA_1c_ at 6 months (−0.6%, 95% CI −1.4% to 0.2% or −6.9 mmol/mol, 95% CI −15.7 to 1.9 mmol/mol; *P*=.12).

A sensitivity analysis excluding participants who never participated in the intervention showed that participants in the digital DSMES+CGM condition had significantly lower HbA_1c_ than those in usual care at both 3 months (−1%, 95% CI −1.6% to −0.3% or −10.5 mmol/mol, 95% CI −17.9 to −3.1 mmol/mol; *P*=.01) and 6 months (−0.8%, 95% CI −1.6% to −0.02% or −9.1 mmol/mol, 95% CI −18.0 to −0.2 mmol/mol; *P*=.046).

### Secondary Outcomes

**Figure 3.**
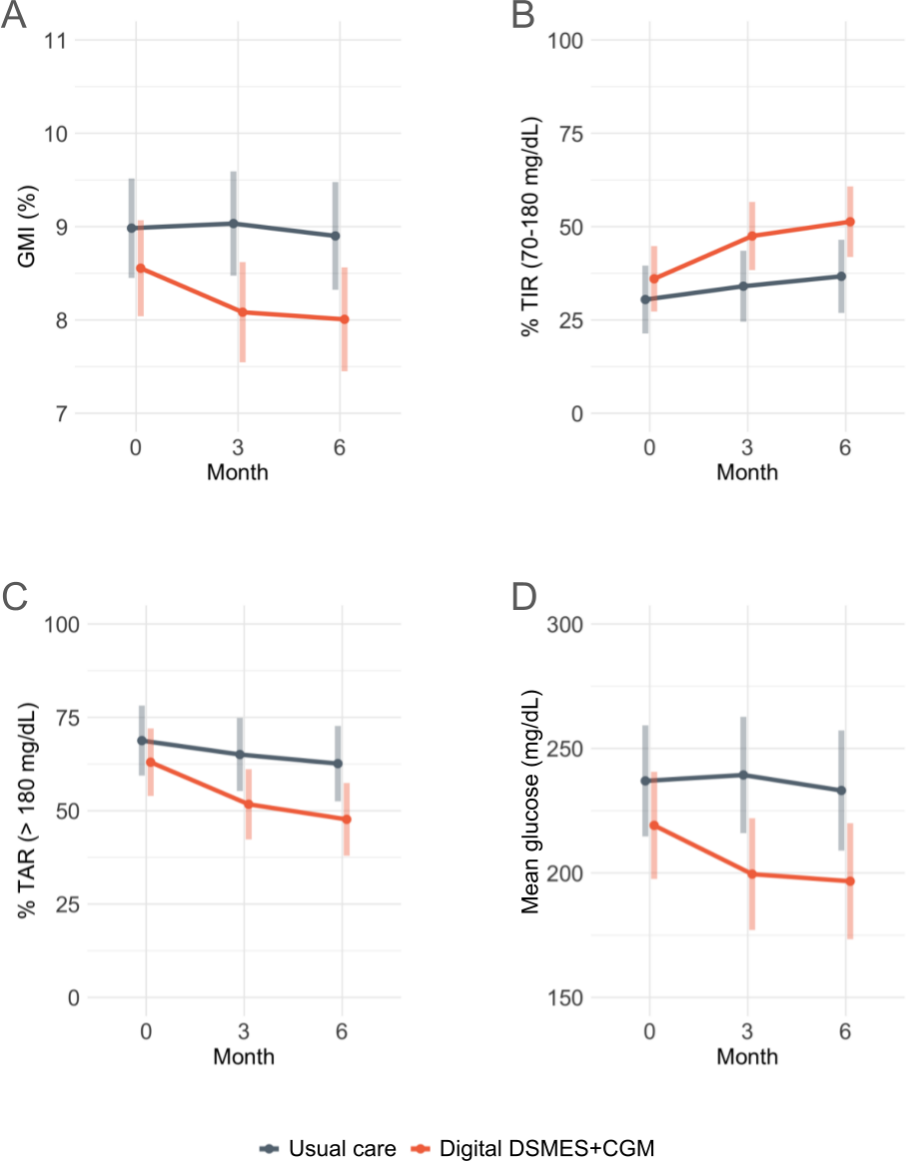
Mean change in (A) GMI (%); (B) % TIR; 70‐180 mg/dL, (C) % TAR; >180 mg/dL, and (D) mean glucose (mg/dL) over time by condition. CGM: continuous glucose monitoring; DSMES: diabetes self-management education and support; GMI: glucose management indicator; TAR: time above range; TIR: time in range.

The results for the secondary outcomes are presented in Table 2 (3-month outcomes), Table 3 (6-month outcomes), and Figure 3. CGM-derived glycemic measures were collected using the blinded FSL Pro sensors. At 3 months, participants in the digital DSMES+CGM condition had a 13.5% (95% CI 0.3% to 26.6%; *P*=.045) higher TIR and a 16.9% (95% CI −30.4% to −3.3%; *P*=.02) lower TAR >250 mg/dL than those in usual care. In addition, at 3 months, participants in the digital DSMES+CGM condition had 39.8 mg/dL (95% CI −72.2 to −7.4 mg/dL; *P*=.02) lower mean glucose and 0.9% (95% CI −1.7% to −0.2%; *P*=.02) lower GMI than those in usual care.

**Table 2.** Secondary clinical outcomes by condition at 3 months.

Variable	Digital DSMES^a^+CGM^b^ (n=51), mean (SE)	Usual care (n=49), mean (SE)	Adjusted mean difference (95% CI)	*P* value^c^
% TIR^d^ (70‐180 mg/dL)	47.5 (4.6)	34.0 (4.8)	13.5 (0.3 to 26.6)	*.045*
% TAR^e^ (>180 mg/dL)	51.7 (4.8)	65.1 (5.0)	−13.3 (−26.9 to 0.2)	.05
% TAR (>250 mg/dL)	23.7 (4.8)	40.6 (5.0)	−16.9 (−30.4 to −3.3)	*.02*
% TBR^f^ (<70 mg/dL)	0.7 (0.4)	0.8 (0.4)	−0.1 (−1.2 to 0.9)	.78
Mean glucose (mg/dL)	199.5 (11.4)	239.4 (11.8)	−39.8 (−72.2 to −7.4)	*.02*
Mean SD of glucose (mg/dL)	40.0 (1.7)	44.4 (1.8)	−4.5 (−9.4 to 0.5)	.08
Mean % CV^g^	21.0 (0.9)	20.1 (0.9)	0.9 (−1.54 to 3.3)	.47
GMI^h^ (%)	8.1 (0.3)	9.0 (0.3)	−0.9 (−1.7 to −0.2)	*.02*
Weight (lb)	212.4 (6.6)	214.7 (6.7)	−2.3 (−21.0 to 16.4)	.81
Systolic BP^i^ (mm Hg)	129.5 (2.3)	130.6 (2.4)	−1.1 (−7.6 to 5.5)	.75
Diastolic BP (mm Hg)	83.7 (1.5)	83.5 (1.5)	0.2 (−3.9 to 4.3)	.92
Diabetes distress	0.8 (0.1)	0.9 (0.1)	−0.1 (−0.3 to 0.1)	.19

a DSMES: diabetes self-management education and support.

b CGM: continuous glucose monitoring.

c *P* values <.05 are italicized.

d TIR: time in range.

e TAR: time above range.

f TBR: time below range.

g CV: coefficient of variation.

h GMI: glucose management indicator.

i BP: blood pressure.

**Table 3.** Secondary clinical outcomes by condition at 6 months.

Variable	Digital DSMES^a^+CGM^b^ (n=51), mean (SE)	Usual care (n=49), mean (SE)	Adjusted mean difference (95% CI)	*P* value^c^
% TIR^d^ (70‐180 mg/dL)	51.3 (4.8)	36.7 (5.0)	14.6 (1.0 to 28.2)	*.04*
% TAR^e^ (>180 mg/dL)	47.7 (4.9)	62.6 (5.1)	−14.9 (−29.0 to −0.9)	*.04*
% TAR (>250 mg/dL)	19.7 (5.0)	37.6 (5.1)	−17.9 (−32.0 to −3.8)	*.01*
% TBR^f^ (<70 mg/dL)	0.9 (0.4)	0.6 (0.4)	0.3 (−0.8 to 1.4)	.60
Mean glucose (mg/dL)	196.7 (11.8)	233.1 (12.2)	−36.4 (−70.0 to −2.9)	*.03*
Mean SD of glucose (mg/dL)	38.9 (1.8)	44.1 (1.9)	−5.2 (−10.3 to −0.1)	*.047*
Mean % CV^g^	21.3 (0.9)	20.6 (0.9)	0.7 (−1.8 to 3.2)	.59
GMI^h^ (%)	8.0 (0.3)	8.9 (0.3)	−0.9 (−1.7 to −0.1)	*.03*
Weight (lb)	212.1 (6.6)	210.6 (6.7)	1.5 (−17.2 to 20.2)	.87
Systolic BP^i^ (mm Hg)	123.7 (2.4)	125.6 (2.3)	−1.9 (−8.5 to 4.6)	.56
Diastolic BP (mm Hg)	82.3 (1.5)	85.1 (1.5)	−2.8 (−6.9 to 1.3)	.18
Diabetes distress	0.7 (0.1)	0.8 (0.1)	−0.1 (−0.3 to 0.0)	.14

a DSMES: diabetes self-management education and support.

b CGM: continuous glucose monitoring.

c *P* values <.05 are italicized.

d TIR: time in range.

e TAR: time above range.

f TBR: time below range.

g CV: coefficient of variation.

h GMI: glucose management indicator.

i BP: blood pressure.

At 6 months, participants in the digital DSMES+CGM condition had a 14.6% (95% CI 1.0% to 28.2%; *P*=.04) higher TIR, a −14.9% (95% CI −29.0% to −0.9%; *P*=.04) lower TAR >180 mg/dL, and a 17.9% (95% CI −32.0% to −3.8%; *P*=.01) lower TAR >250 mg/dL than those in usual care. In addition, at 6 months, participants in the digital DSMES+CGM condition had 36.4 mg/dL (95% CI −70.0 to −2.9 mg/dL; *P*=.03) lower mean glucose, 5.2 mg/dL (95% CI −10.3 to −0.01 mg/dL; *P*=.047) lower SD of glucose, and 0.9% (95% CI −1.7% to −0.1%; *P*=.03) lower GMI than those in usual care.

Finally, no statistically significant differences between the intervention and control groups were seen for weight, systolic or diastolic blood pressure, or diabetes distress at either 3 or 6 months.

When conducting sensitivity analyses excluding participants who never enrolled or participated in the intervention, results remained largely the same as the ITT analyses with 2 exceptions: participants in the digital DSMES+CGM condition had significantly lower TAR >180 mg/dL (−16.3%, 95% CI −30.3% to −2.3%; *P*=.02) and SD of glucose (−5.8 mg/dL, 95% CI −11.0 to −0.7 mg/dL; *P*=.03) than those in usual care at 3 months.

There were no reported serious adverse events related to the study devices or program, and the number of adverse events did not differ significantly across conditions.

## Discussion

Results from this RCT evaluating an integrated digital DSMES program+CGM revealed that participants randomized to the integrated solution experienced improvements in HbA_1c_ and CGM-derived measures of glycemic control compared to usual care over 6 months. Though the difference between conditions in HbA_1c_ at 6 months was not significant within an ITT analysis, sensitivity analyses showed that participants who enrolled in the digital DSMES+CGM condition had significantly lower HbA_1c_ than those in usual care. These findings are clinically meaningful given that, on average, the intervention group saw an almost 1-point reduction in HbA_1c_ levels over the study period—a degree of change that is twice as large as the benchmark for a clinically significant change in HbA_1c_ of 0.5% [33] and is associated with a reduced risk of diabetes-related complications, including microvascular disease, myocardial infarction, heart failure, stroke, and death [34].

Given the focus of this trial on the integration of CGM with DSMES [35], CGM-derived glycemic measures also improved in the digital DSMES+CGM condition compared to usual care. From baseline to 6 months, participants in the digital DSMES+CGM condition experienced a 15% increase in percent TIR and a 15% decrease in percent TAR >180 mg/dL, which were more favorable improvements compared to usual care and at a magnitude of change 3-fold greater than what previous research has demonstrated to be clinically meaningful [36]. Additionally, participants in our integrated solution had a 0.89% lower GMI than those in usual care at 6 months, indicating that those receiving the digital DSMES program+CGM had better glycemic control and a lower risk for diabetes-related complications [37]. Together, our findings demonstrate improvement in TIR and reduced variability of blood glucose among participants who received the digital DSMES+CGM integrated solution compared to usual care.

The design of our integrated solution leveraged a combination of the benefits of CGM and DSMES programs for type 2 diabetes management. Previous research revealed that using CGM for 8 weeks helped people with diabetes significantly reduce their HbA_1c_ by an average 1-point reduction, which is consistent with our present study and demonstrates the importance of self-monitoring for diabetes management [38]. Additional research also showed that coupling CGMs with DSMES over 16 weeks led to significantly greater mean TIR, significantly less TAR, and a greater reduction in mean HbA_1c_ compared to DSMES alone [15]. However, a unique feature of our clinical trial is the integration of both real-time CGM and digital DSMES over a longer 6-month period to evaluate the consistent and sustainable aspects of the intervention.

As part of the intervention, research participants in the digital DSMES program received 1:1 support from a CDCES, which included systematic outreach at fixed program weeks in addition to continuous health coaching and tailored feedback in response to members’ biometric data collected throughout the program (eg, CGM glucose values and body weight). Their strategic outreach and continuous monitoring highlights the proactive feature of the integrated solution, aligning well with past research revealing that proactive case management significantly improved glycemic control in patients with diabetes [39]. Furthermore, digital DSMES+CGM participants in our study were also highly engaged, as exhibited by frequent weighing, tracking meals, exercising, and CGM-wearing for several days per week. These behaviors are impactful, given their association with improved glycemic control in the digital DSMES program [26,40].

Our study has several strengths, with a notable advantage being the rigor of its single-blind RCT design. The sensitivity analysis also allowed us to more accurately estimate the impact of the integrated solution on clinical outcomes among participants who had the opportunity to engage with it. The convergence between the ITT and sensitivity results demonstrates the beneficial impact of the digital DSMES program+CGM on clinical outcomes. Furthermore, by leveraging a decentralized recruitment process, we were able to include a racially, socioeconomically, and geographically diverse group of participants. This inclusive, patient-centered approach enhanced convenience and minimized dropout by bringing clinicians to participants’ homes for assessments [41]. Moreover, our trial’s postrandomization attrition rate was 12%, despite having a longitudinal study design that required several follow-up assessments and biospecimen collection. Notably, this attrition rate is lower than the approximate 30% dropout rate typically seen across site-based clinical trials [42], underscoring the benefits of decentralized operations.

We also acknowledge several limitations with the current study. First, the complexities of remote study operations, such as costs, shipping delays, scheduling conflicts, and recruitment difficulties, caused us to fall short of our intended sample size and highlighted the challenges of implementing a decentralized RCT. However, the clinically and statistically significant results observed with a smaller sample size underscore the effectiveness of the digital DSMES+CGM integrated solution. Second, 9 participants randomized to the intervention did not participate in the digital DSMES program and, therefore, did not experience the integrated solution. Reasons contributing to treatment nonadherence included screen failure due to disqualifying TIR or previous exposure to the DSMES program, failure to apply to the DSMES program, and eventual loss to follow-up. That said, all 9 participants had at least 1 follow-up assessment, so we were still able to evaluate their clinical outcomes. Finally, as part of the integrated solution, participants were expected to wear an FSL 14-Day sensor continuously for the duration of the study (along with 2 FSL Pro sensors at baseline, 3 mo, and 6 mo for measurement purposes), which may have increased participant burden and impacted adherence over the 6-month study period. However, these results likely reflect real-world compliance with CGM usage on a long-term, continuous basis.

In conclusion, the statistically significant and clinically meaningful improvement in HbA_1c_ and CGM outcomes among participants in the digital DSMES+CGM group demonstrates that combining CGMs with a digital DSMES program can have a beneficial impact on glucose management among adults with type 2 diabetes. Nevertheless, longer and larger-scale trials are warranted to assess the reliability of our findings and the benefit of our integrated solution on potentially reducing the risk of long-term health complications. Ultimately, increasing access to digital DSMES programs with integrated CGM capabilities may be a promising approach to help adults with type 2 diabetes manage their condition, improve health outcomes, and support broader efforts to enhance the quality and effectiveness of diabetes care.

## Supplementary material

10.2196/78321 Checklist 1 CONSORT-EHEALTH (V 1.6.1) checklist.

## Abbreviations

CDCES: Certified Diabetes Care and Education Specialist
CGM: continuous glucose monitoring
DSMES: diabetes self-management education and support
FSL: FreeStyle Libre
GMI: glucose management indicator
HbA_1c_: hemoglobin A_1c_
ITT: intention-to-treat
MCAR: missing completely at random
RCT: randomized controlled trial
TAR: time above range
TBR: time below range
TIR: time in range
