# Supplementary material for: A Digital Diabetes Self-Management Education and Support Program Integrated With Continuous Glucose Monitoring for Type 2 Diabetes: Randomized Controlled Trial
Source: J Med Internet Res. 2026 May 14;28:e78321. doi: 10.2196/78321 (PMC13175446; doi:10.2196/78321)
Supplement: Checklist 1 [file jmir-v28-e78321-s001.pdf]

CONSORT-EHEALTH (V 1.6.1) - Submission/Publication Form

The CONSORT-EHEALTH checklist is intended for authors of randomized trials evaluating web-based and Internet-based applications/interventions, including mobile interventions, electronic games (incl multiplayer games), social media, certain telehealth applications, and other interactive and/or networked electronic applications. Some of the items (e.g. all subitems under item 5 - description of the intervention) may also be applicable for other study designs.

The goal of the CONSORT EHEALTH checklist and guideline is to be
a) a guide for reporting for authors of RCTs,
b) to form a basis for appraisal of an ehealth trial (in terms of validity)

CONSORT-EHEALTH items/subitems are MANDATORY reporting items for studies published in the Journal of Medical Internet Research and other journals / scientific societies endorsing the checklist.

Items numbered 1., 2., 3., 4a., 4b etc are original CONSORT or CONSORT-NPT (non-pharmacologic treatment) items.
Items with Roman numerals (i., ii, iii, iv etc.) are CONSORT-EHEALTH extensions/clarifications.

As the CONSORT-EHEALTH checklist is still considered in a formative stage, we would ask that you also RATE ON A SCALE OF 1-5 how important/useful you feel each item is FOR THE PURPOSE OF THE CHECKLIST and reporting guideline (optional).

Mandatory reporting items are marked with a red *.
In the textboxes, either copy & paste the relevant sections from your manuscript into this form - please include any quotes from your manuscript in QUOTATION MARKS,
or answer directly by providing additional information not in the manuscript, or elaborating on why the item was not relevant for this study.

YOUR ANSWERS WILL BE PUBLISHED AS A SUPPLEMENTARY FILE TO YOUR PUBLICATION IN JMIR AND ARE CONSIDERED PART OF YOUR PUBLICATION (IF ACCEPTED).
Please fill in these questions diligently. Information will not be copyedited, so please use proper spelling and grammar, use correct capitalization, and avoid abbreviations.

DO NOT FORGET TO SAVE AS PDF _AND_ CLICK THE SUBMIT BUTTON SO YOUR ANSWERS ARE IN OUR DATABASE !!!

Citation Suggestion (if you append the pdf as Appendix we suggest to cite this paper in the caption):
Eysenbach G, CONSORT-EHEALTH Group

CONSORT-EHEALTH: Improving and Standardizing Evaluation Reports of Web-based and Mobile Health Interventions
J Med Internet Res 2011;13(4):e126
URL: http://www.jmir.org/2011/4/e126/
doi: 10.2196/jmir.1923
PMID: 22209829

jenna.napoleone@omadahealth.com Switch account

Draft not saved

Not shared

* Indicates required question

Your name *

First Last

Jenna Napoleone

Primary Affiliation (short), City, Country *

University of Toronto, Toronto, Canada

Omada Health

Your e-mail address *

abc@gmail.com

jenna.napoleone@omadahealth.com

Title of your manuscript *

Provide the (draft) title of your manuscript.

A Digital Diabetes Self-Management Education and Support Program Integrated With Continuous Glucose Monitoring Improves Glycemic Control: A Randomized Controlled Trial

Name of your App/Software/Intervention *

If there is a short and a long/alternate name, write the short name first and add the long name in brackets.

Omada for Diabetes program

Evaluated Version (if any)

e.g. "V1", "Release 2017-03-01", "Version 2.0.27913"

Your answer

Language(s) *

What language is the intervention/app in? If multiple languages are available, separate by comma (e.g. "English, French")

English

URL of your Intervention Website or App

e.g. a direct link to the mobile app on app in appstore (itunes, Google Play), or URL of the website. If the intervention is a DVD or hardware, you can also link to an Amazon page.

https://www.omadahealth.com/

URL of an image/screenshot (optional)

Your answer

Accessibility *

Can an enduser access the intervention presently?

- ( ) access is free and open
- (x) access only for special usergroups, not open
- ( ) access is open to everyone, but requires payment/subscription/in-app purchases
- ( ) app/intervention no longer accessible
- ( ) Other:

Primary Medical Indication/Disease/Condition *

e.g. "Stress", "Diabetes", or define the target group in brackets after the condition, e.g. "Autism (Parents of children with)", "Alzheimers (Informal Caregivers of)"

Type 2 Diabetes

Primary Outcomes measured in trial *

comma-separated list of primary outcomes reported in the trial

"The primary outcome was HbA1c level."

Secondary/other outcomes

Are there any other outcomes the intervention is expected to affect?

“Secondary outcomes measured via the FSL Pro sensors included CGM 14-day % TIR, % time above range (TAR), and % time below range (TBR). Furthermore, differences in mean blood glucose, standard deviation (SD) of blood glucose, coefficient of variation, and glucose management indicator (GMI) were examined. Lastly, body weight, resting systolic and diastolic blood pressure, and diabetes distress27 were examined.”

Recommended "Dose" *

What do the instructions for users say on how often the app should be used?

- ( ) Approximately Daily
- ( ) Approximately Weekly
- ( ) Approximately Monthly
- ( ) Approximately Yearly
- ( ) "as needed"
- (•) Other: Users can interact with the app as frequently as they desire.

Approx. Percentage of Users (starters) still using the app as recommended after 3 months *

- (•) unknown / not evaluated
- ( ) 0-10%
- ( ) 11-20%
- ( ) 21-30%
- ( ) 31-40%
- ( ) 41-50%
- ( ) 51-60%
- ( ) 61-70%
- ( ) 71%-80%
- ( ) 81-90%
- ( ) 91-100%
- ( ) Other:

Overall, was the app/intervention effective? *

- ( ) yes: all primary outcomes were significantly better in intervention group vs control
- (•) partly: SOME primary outcomes were significantly better in intervention group vs control
- ( ) no statistically significant difference between control and intervention
- ( ) potentially harmful: control was significantly better than intervention in one or more outcomes
- ( ) inconclusive: more research is needed
- ( ) Other:

Article Preparation Status/Stage *

At which stage in your article preparation are you currently (at the time you fill in this form)

- ( ) not submitted yet - in early draft status
- (•) not submitted yet - in late draft status, just before submission
- ( ) submitted to a journal but not reviewed yet
- ( ) submitted to a journal and after receiving initial reviewer comments
- ( ) submitted to a journal and accepted, but not published yet
- ( ) published
- ( ) Other:

Journal *

If you already know where you will submit this paper (or if it is already submitted), please provide the journal name (if it is not JMIR, provide the journal name under "other")

- ◯ not submitted yet / unclear where I will submit this
- ◯ Journal of Medical Internet Research (JMIR)
- ◯ JMIR mHealth and UHealth
- ◯ JMIR Serious Games
- ◯ JMIR Mental Health
- ◯ JMIR Public Health
- ◯ JMIR Formative Research
- ◯ Other JMIR sister journal
- ◉ Other: JMIR Diabetes

Is this a full powered effectiveness trial or a pilot/feasibility trial? *

- ◯ Pilot/feasibility
- ◉ Fully powered

Manuscript tracking number *

If this is a JMIR submission, please provide the manuscript tracking number under "other" (The ms tracking number can be found in the submission acknowledgement email, or when you login as author in JMIR. If the paper is already published in JMIR, then the ms tracking number is the four-digit number at the end of the DOI, to be found at the bottom of each published article in JMIR)

- ◉ no ms number (yet) / not (yet) submitted to / published in JMIR
- ◯ Other:

TITLE AND ABSTRACT

1a) TITLE: Identification as a randomized trial in the title

1a) Does your paper address CONSORT item 1a? *

I.e does the title contain the phrase "Randomized Controlled Trial"? (if not, explain the reason under "other")

- (●) yes
- ( ) Other:

1a-i) Identify the mode of delivery in the title

Identify the mode of delivery. Preferably use “web-based” and/or “mobile” and/or “electronic game” in the title. Avoid ambiguous terms like “online”, “virtual”, “interactive”. Use “Internet-based” only if Intervention includes non-web-based Internet components (e.g. email), use “computer-based” or “electronic” only if offline products are used. Use “virtual” only in the context of “virtual reality” (3-D worlds). Use “online” only in the context of “online support groups”. Complement or substitute product names with broader terms for the class of products (such as “mobile” or “smart phone” instead of “iphone”), especially if the application runs on different platforms.

	1	2	3	4	5	
subitem not at all important	○	○	○	○	○	essential

Does your paper address subitem 1a-i? *

Copy and paste relevant sections from manuscript title (include quotes in quotation marks "like this" to indicate direct quotes from your manuscript), or elaborate on this item by providing additional information not in the ms, or briefly explain why the item is not applicable/relevant for your study

"A Digital Diabetes Self-Management Education and Support Program Integrated With Continuous Glucose Monitoring Improves Glycemic Control: A Randomized Controlled Trial "

1a-ii) Non-web-based components or important co-interventions in title

Mention non-web-based components or important co-interventions in title, if any (e.g., “with telephone support”).

	1	2	3	4	5	
subitem not at all important	○	○	○	○	○	essential

Does your paper address subitem 1a-ii?

Copy and paste relevant sections from manuscript title (include quotes in quotation marks "like this" to indicate direct quotes from your manuscript), or elaborate on this item by providing additional information not in the ms, or briefly explain why the item is not applicable/relevant for your study

A Digital Diabetes Self-Management Education and Support Program Integrated With Continuous Glucose Monitoring Improves Glycemic Control: A Randomized Controlled Trial

1a-iii) Primary condition or target group in the title

Mention primary condition or target group in the title, if any (e.g., “for children with Type I Diabetes”) Example: A Web-based and Mobile Intervention with Telephone Support for Children with Type I Diabetes: Randomized Controlled Trial

	1	2	3	4	5	
subitem not at all important	○	○	○	○	○	essential

Does your paper address subitem 1a-iii? *

Copy and paste relevant sections from manuscript title (include quotes in quotation marks "like this" to indicate direct quotes from your manuscript), or elaborate on this item by providing additional information not in the ms, or briefly explain why the item is not applicable/relevant for your study

"A Digital Diabetes Self-Management Education and Support Program"

1b) ABSTRACT: Structured summary of trial design, methods, results, and conclusions

NPT extension: Description of experimental treatment, comparator, care providers, centers, and blinding status.

1b-i) Key features/functionalities/components of the intervention and comparator in the METHODS section of the ABSTRACT

Mention key features/functionalities/components of the intervention and comparator in the abstract. If possible, also mention theories and principles used for designing the site. Keep in mind the needs of systematic reviewers and indexers by including important synonyms. (Note: Only report in the abstract what the main paper is reporting. If this information is missing from the main body of text, consider adding it)

	1	2	3	4	5	
subitem not at all important	○	○	○	○	○	essential

Does your paper address subitem 1b-i? *

Copy and paste relevant sections from the manuscript abstract (include quotes in quotation marks "like this" to indicate direct quotes from your manuscript), or elaborate on this item by providing additional information not in the ms, or briefly explain why the item is not applicable/relevant for your study

"Participants with type 2 diabetes and HbA1c ≥8% not using mealtime bolus insulin (26–83 years old; mean HbA1c: 9.58% [81 mmol/mol]) were randomly assigned to a digital DSMES + CGM integrated solution (n=51) or usual care (n=49) for 6 months."

1b-ii) Level of human involvement in the METHODS section of the ABSTRACT

Clarify the level of human involvement in the abstract, e.g., use phrases like “fully automated” vs. “therapist/nurse/care provider/physician-assisted” (mention number and expertise of providers involved, if any). (Note: Only report in the abstract what the main paper is reporting. If this information is missing from the main body of text, consider adding it)

	1	2	3	4	5	
subitem not at all important	○	○	○	○	○	essential

Does your paper address subitem 1b-ii?

Copy and paste relevant sections from the manuscript abstract (include quotes in quotation marks "like this" to indicate direct quotes from your manuscript), or elaborate on this item by providing additional information not in the ms, or briefly explain why the item is not applicable/relevant for your study

Your answer

1b-iii) Open vs. closed, web-based (self-assessment) vs. face-to-face assessments in the METHODS section of the ABSTRACT

Mention how participants were recruited (online vs. offline), e.g., from an open access website or from a clinic or a closed online user group (closed usergroup trial), and clarify if this was a purely web-based trial, or there were face-to-face components (as part of the intervention or for assessment). Clearly say if outcomes were self-assessed through questionnaires (as common in web-based trials). Note: In traditional offline trials, an open trial (open-label trial) is a type of clinical trial in which both the researchers and participants know which treatment is being administered. To avoid confusion, use “blinded” or “unblinded” to indicated the level of blinding instead of “open”, as “open” in web-based trials usually refers to “open access” (i.e. participants can self-enrol). (Note: Only report in the abstract what the main paper is reporting. If this information is missing from the main body of text, consider adding it)

	1	2	3	4	5	
subitem not at all important	○	○	○	○	○	essential

Does your paper address subitem 1b-iii?

Copy and paste relevant sections from the manuscript abstract (include quotes in quotation marks "like this" to indicate direct quotes from your manuscript), or elaborate on this item by providing additional information not in the ms, or briefly explain why the item is not applicable/relevant for your study

Your answer

1b-iv) RESULTS section in abstract must contain use data

Report number of participants enrolled/assessed in each group, the use/uptake of the intervention (e.g., attrition/adherence metrics, use over time, number of logins etc.), in addition to primary/secondary outcomes. (Note: Only report in the abstract what the main paper is reporting. If this information is missing from the main body of text, consider adding it)

	1	2	3	4	5	
subitem not at all important	○	○	○	○	○	essential

Does your paper address subitem 1b-iv?

Copy and paste relevant sections from the manuscript abstract (include quotes in quotation marks "like this" to indicate direct quotes from your manuscript), or elaborate on this item by providing additional information not in the ms, or briefly explain why the item is not applicable/relevant for your study

Your answer

1b-v) CONCLUSIONS/DISCUSSION in abstract for negative trials

Conclusions/Discussions in abstract for negative trials: Discuss the primary outcome - if the trial is negative (primary outcome not changed), and the intervention was not used, discuss whether negative results are attributable to lack of uptake and discuss reasons. (Note: Only report in the abstract what the main paper is reporting. If this information is missing from the main body of text, consider adding it)

	1	2	3	4	5	
subitem not at all important	○	○	○	○	○	essential

Does your paper address subitem 1b-v?

Copy and paste relevant sections from the manuscript abstract (include quotes in quotation marks "like this" to indicate direct quotes from your manuscript), or elaborate on this item by providing additional information not in the ms, or briefly explain why the item is not applicable/relevant for your study

Your answer

INTRODUCTION

2a) In INTRODUCTION: Scientific background and explanation of rationale

2a-i) Problem and the type of system/solution

Describe the problem and the type of system/solution that is object of the study: intended as stand-alone intervention vs. incorporated in broader health care program? Intended for a particular patient population? Goals of the intervention, e.g., being more cost-effective to other interventions, replace or complement other solutions? (Note: Details about the intervention are provided in “Methods” under 5)

	1	2	3	4	5	
subitem not at all important	○	○	○	○	○	essential

Does your paper address subitem 2a-i? *

Copy and paste relevant sections from the manuscript (include quotes in quotation marks "like this" to indicate direct quotes from your manuscript), or elaborate on this item by providing additional information not in the ms, or briefly explain why the item is not applicable/relevant for your study

"Despite the growing observational research demonstrating the effectiveness of digital DSMES and CGM, to date, no RCTs have shown the benefits of a well-integrated solution that combines a digitally-delivered DSMES program with longer-term, real-time, continuous CGM for adults living with type 2 diabetes"

2a-ii) Scientific background, rationale: What is known about the (type of) system

Scientific background, rationale: What is known about the (type of) system that is the object of the study (be sure to discuss the use of similar systems for other conditions/diagnoses, if appropiate), motivation for the study, i.e. what are the reasons for and what is the context for this specific study, from which stakeholder viewpoint is the study performed, potential impact of findings [2]. Briefly justify the choice of the comparator.

	1	2	3	4	5	
subitem not at all important	○	○	○	○	○	essential

Does your paper address subitem 2a-ii? *

Copy and paste relevant sections from the manuscript (include quotes in quotation marks "like this" to indicate direct quotes from your manuscript), or elaborate on this item by providing additional information not in the ms, or briefly explain why the item is not applicable/relevant for your study

"However, previous research evaluating DSMES programs integrated with CGM has predominantly focused on in-person programs and short-term intermittent CGM use for less than 8 weeks.13–16 A few observational studies have explored the effects of a telemedicine lifestyle program for diabetes that included a CGM offering,17,18 but CGM use among participants was short-term and the findings could not be extrapolated to show a cause-effect relationship between the virtually integrated approach and improved glycemic control.
Nevertheless, a meta-analysis of randomized controlled trials (RCTs) evaluating the clinical effectiveness of telemedicine for diabetes revealed that virtual healthcare services are generally more effective for diabetes care management than in-person care alone.19 People participating in digital diabetes care programs have seen improved clinical outcomes (e.g., HbA1c reduction) and reduced medical expenditures,20–22 but clinical trials evaluating many of these digital programs have not reported CGM data related to their interventions for type 2 diabetes.23–25"

2b) In INTRODUCTION: Specific objectives or hypotheses

Does your paper address CONSORT subitem 2b? *

Copy and paste relevant sections from the manuscript (include quotes in quotation marks "like this" to indicate direct quotes from your manuscript), or elaborate on this item by providing additional information not in the ms, or briefly explain why the item is not applicable/relevant for your study

"Therefore, the aim of this RCT was to evaluate the impact of the integrated solution—defined as a digital DSMES program + CGM—on HbA1c, CGM-derived glycemic measures, and other relevant diabetes management outcomes compared to usual care over 6 months."

METHODS

3a) Description of trial design (such as parallel, factorial) including allocation ratio

Does your paper address CONSORT subitem 3a? *

Copy and paste relevant sections from the manuscript (include quotes in quotation marks "like this" to indicate direct quotes from your manuscript), or elaborate on this item by providing additional information not in the ms, or briefly explain why the item is not applicable/relevant for your study

"This single-blind, parallel decentralized RCT "

3b) Important changes to methods after trial commencement (such as eligibility criteria), with reasons

Does your paper address CONSORT subitem 3b? *

Copy and paste relevant sections from the manuscript (include quotes in quotation marks "like this" to indicate direct quotes from your manuscript), or elaborate on this item by providing additional information not in the ms, or briefly explain why the item is not applicable/relevant for your study

"During the initial recruitment period (June-October 2022), participants were also required to have a percent time in range (TIR) between 70-180 mg/dL of <60% prior to enrollment, which was eventually removed from the eligibility criteria to reduce participant burden and improve study activity workflow during the remainder of the recruitment period (October 2022-May 2023)."

3b-i) Bug fixes, Downtimes, Content Changes

Bug fixes, Downtimes, Content Changes: ehealth systems are often dynamic systems. A description of changes to methods therefore also includes important changes made on the intervention or comparator during the trial (e.g., major bug fixes or changes in the functionality or content) (5-iii) and other “unexpected events” that may have influenced study design such as staff changes, system failures/downtimes, etc. [2].

	1	2	3	4	5	
subitem not at all important	○	○	○	○	○	essential

Does your paper address subitem 3b-i?

Copy and paste relevant sections from the manuscript (include quotes in quotation marks "like this" to indicate direct quotes from your manuscript), or elaborate on this item by providing additional information not in the ms, or briefly explain why the item is not applicable/relevant for your study

Your answer

4a) Eligibility criteria for participants

Does your paper address CONSORT subitem 4a? *

Copy and paste relevant sections from the manuscript (include quotes in quotation marks "like this" to indicate direct quotes from your manuscript), or elaborate on this item by providing additional information not in the ms, or briefly explain why the item is not applicable/relevant for your study

"To meet inclusion criteria, participants had to be at least 18 years old, living in the United States, and have a self-reported type 2 diabetes diagnosis for at least 6 months prior to enrollment. Additionally, they needed a laboratory-confirmed HbA1c level of ≥8% (64 mmol/mol) at baseline and a treatment regimen that included a combination of basal insulin, oral medications, and/or diet and exercise. During the initial recruitment period (June-October 2022), participants were also required to have a percent time in range (TIR) between 70-180 mg/dL of <60% prior to enrollment, which was eventually removed from the eligibility criteria to reduce participant burden and improve study activity workflow during the remainder of the recruitment period (October 2022-May 2023).
Exclusion criteria included type 1 diabetes or a history of diabetic ketoacidosis, mealtime bolus insulin therapy, premixed insulin formulations or a continuous subcutaneous infusion of insulin, routine regimen of glucocorticoids or psychotropic medications, bariatric surgery or organ transplant within 6 months before enrollment, and pregnancy or intentions of conceiving during the study period (female participants). See Supplemental Figure S1 for more details on participant flow through the study.
"
"Those who passed an initial screening proceeded to sign an electronic informed consent form for the study and complete an online baseline survey, which collected their demographic information, health and diabetes history, and patient-reported outcomes. A secondary screening visit was performed to confirm that potential participants met the additional eligibility criteria of an HbA1c level of ≥8% (64 mmol/mol) and, prior to October 2022, a TIR (70-180 mg/dL) of <60%. Contracted vendors (PCM Trials/Act for Health, Inc. and Hawthorne Effect, Inc.) deployed certified and trained clinicians to conduct home visits in cities and rural areas across the US, eliminating the need for participants to travel to a physical site or clinic for clinical assessments. During the home visits, they collected up to 4 mL of blood via venipuncture, anthropometric measurements, resting blood pressure, and a list of participants' cardiometabolic medications. They also applied and activated two FSL Pro CGM sensors on the participants for measurement purposes, and participants were instructed to wear the FSL Pro CGM sensors for up to 14 days while blinded to the data. Afterwards, participants mailed the devices to the study team for data download and TIR analysis. The contracted clinicians mailed the blood specimens to Quest Diagnostics laboratory for HbA1c analysis. Participants who had a laboratory-confirmed HbA1c ≥8.0% (64 mmol/mol), and a TIR (70-180 mg/dL) of <60% if recruited prior to mid-October 2022, were randomized and considered enrolled in the study."

4a-i) Computer / Internet literacy

Computer / Internet literacy is often an implicit “de facto” eligibility criterion - this should be explicitly clarified.

	1	2	3	4	5	
subitem not at all important	○	○	○	○	○	essential

Does your paper address subitem 4a-i?

Copy and paste relevant sections from the manuscript (include quotes in quotation marks "like this" to indicate direct quotes from your manuscript), or elaborate on this item by providing additional information not in the ms, or briefly explain why the item is not applicable/relevant for your study

Your answer

4a-ii) Open vs. closed, web-based vs. face-to-face assessments:

Open vs. closed, web-based vs. face-to-face assessments: Mention how participants were recruited (online vs. offline), e.g., from an open access website or from a clinic, and clarify if this was a purely web-based trial, or there were face-to-face components (as part of the intervention or for assessment), i.e., to what degree got the study team to know the participant. In online-only trials, clarify if participants were quasi-anonymous and whether having multiple identities was possible or whether technical or logistical measures (e.g., cookies, email confirmation, phone calls) were used to detect/prevent these.

	1	2	3	4	5	
subitem not at all important	○	○	○	○	○	essential

Does your paper address subitem 4a-ii? *

Copy and paste relevant sections from the manuscript (include quotes in quotation marks "like this" to indicate direct quotes from your manuscript), or elaborate on this item by providing additional information not in the ms, or briefly explain why the item is not applicable/relevant for your study

"We recruited individuals to join this decentralized trial through Evidation (Evidation Health, Inc., San Mateo, CA), an online health platform through which users connect their digital health tools, such as fitness apps and wearable activity trackers, in exchange for monetary incentives. Targeted emails and platform posts were sent to existing Evidation members who agreed to receive communications about studies that may be relevant to them based on health and lifestyle information they have provided to the platform. We also recruited non-Evidation members via advertisements on social media and online forums. " "Those who passed an initial screening proceeded to sign an electronic informed consent form for the study and complete an online baseline survey, which collected their demographic information, health and diabetes history, and patient-reported outcomes. A secondary screening visit was performed to confirm that potential participants met the additional eligibility criteria of an HbA1c level of ≥8% (64 mmol/mol) and, prior to October 2022, a TIR (70-180 mg/dL) of <60%. Contracted vendors (PCM Trials/Act for Health, Inc. and Hawthorne Effect, Inc.) deployed certified and trained clinicians to conduct home visits in cities and rural areas across the US, eliminating the need for participants to travel to a physical site or clinic for clinical assessments. During the home visits, they collected up to 4 mL of blood via venipuncture, anthropometric measurements, resting blood pressure, and a list of participants' cardiometabolic medications. They also applied and activated two FSL Pro CGM sensors on the participants for measurement purposes, and participants were instructed to wear the FSL Pro CGM sensors for up to 14 days while blinded to the data. Afterwards, participants mailed the devices to the study team for data download and TIR analysis. The contracted clinicians mailed the blood specimens to Quest Diagnostics laboratory for HbA1c analysis. Participants who had a laboratory-confirmed HbA1c ≥8.0% (64 mmol/mol), and a TIR (70-180 mg/dL) of <60% if recruited prior to mid-October 2022, were randomized and considered enrolled in the study."
"The assessment staff members (i.e., clinicians conducting home visits, Quest Diagnostics) and the Evidation-designated statistician that developed the statistical analysis plan were blinded to the intervention assignment during the study period. Adverse-event adjudicators, designated research personnel at Evidation and Omada, as well as Omada coaching staff, were not blinded. Participants were not blinded to their assignment, but they were blinded to their HbA1c levels and CGM blood glucose values collected via FSL Pro sensors."

4a-iii) Information giving during recruitment

Information given during recruitment. Specify how participants were briefed for recruitment and in the informed consent procedures (e.g., publish the informed consent documentation as appendix, see also item X26), as this information may have an effect on user self-selection, user expectation and may also bias results.

	1	2	3	4	5	
subitem not at all important	○	○	○	○	○	essential

Does your paper address subitem 4a-iii?

Copy and paste relevant sections from the manuscript (include quotes in quotation marks "like this" to indicate direct quotes from your manuscript), or elaborate on this item by providing additional information not in the ms, or briefly explain why the item is not applicable/relevant for your study

Your answer

4b) Settings and locations where the data were collected

Does your paper address CONSORT subitem 4b? *

Copy and paste relevant sections from the manuscript (include quotes in quotation marks "like this" to indicate direct quotes from your manuscript), or elaborate on this item by providing additional information not in the ms, or briefly explain why the item is not applicable/relevant for your study

"Those who passed an initial screening proceeded to sign an electronic informed consent form for the study and complete an online baseline survey, which collected their demographic information, health and diabetes history, and patient-reported outcomes. A secondary screening visit was performed to confirm that potential participants met the additional eligibility criteria of an HbA1c level of ≥8% (64 mmol/mol) and, prior to October 2022, a TIR (70-180 mg/dL) of <60%. Contracted vendors (PCM Trials/Act for Health, Inc. and Hawthorne Effect, Inc.) deployed certified and trained clinicians to conduct home visits in cities and rural areas across the US, eliminating the need for participants to travel to a physical site or clinic for clinical assessments. During the home visits, they collected up to 4 mL of blood via venipuncture, anthropometric measurements, resting blood pressure, and a list of participants' cardiometabolic medications. They also applied and activated two FSL Pro CGM sensors on the participants for measurement purposes, and participants were instructed to wear the FSL Pro CGM sensors for up to 14 days while blinded to the data. Afterwards, participants mailed the devices to the study team for data download and TIR analysis. The contracted clinicians mailed the blood specimens to Quest Diagnostics laboratory for HbA1c analysis. Participants who had a laboratory-confirmed HbA1c ≥8.0% (64 mmol/mol), and a TIR (70-180 mg/dL) of <60% if recruited prior to mid-October 2022, were randomized and considered enrolled in the study.
Randomization and Masking
Each eligible participant was randomized to one of the two conditions (digital DSMES + CGM or usual care) according to an Evidation-controlled algorithm that used randomly permuted blocks of fixed size. Randomization was stratified by gender to ensure nearly equal sample sizes for the two treatment arms for each gender.
The assessment staff members (i.e., clinicians conducting home visits, Quest Diagnostics) and the Evidation-designated statistician that developed the statistical analysis plan were blinded to the intervention assignment during the study period. Adverse-event adjudicators, designated research personnel at Evidation and Omada, as well as Omada coaching staff, were not blinded. Participants were not blinded to their assignment, but they were blinded to their HbA1c levels and CGM blood glucose values collected via FSL Pro sensors.
"
"Participants randomized to the digital DSMES + CGM intervention arm completed an application for the Omada for Diabetes program, a digitally-delivered DSMES program that has achieved accreditation from the National Committee for Quality Assurance’s Population Health Program and Accreditation and the Association of Diabetes Care and Education Specialists. The program pairs asynchronous health coaching from a care team comprised of certified Diabetes Prevention Program Lifestyle Coaches and Certified Diabetes Care and Education Specialists (CDCESs) with a virtual platform accessed via website or mobile app available on web-enabled devices. Upon enrollment, members receive cellularly-connected FDA devices, such as body weight scales and CGMs, along with tailored diabetes self-management and lifestyle modification support under the guidance of their care team. The care team interacts with members asynchronously through messaging on the virtual platform, regularly monitoring participants’ engagement and biometric data collected during the program and using it to provide tailored feedback on diabetes self-management through proactive outreach and/or responding to member questions. Additional program features include weekly lessons with nutrition and lifestyle content, support around personalized goal setting, and online peer communities.26
After submitting an application to join the digital DSMES program as research members, digital DSMES + CGM participants completed their account setup through a one-time [illegible]

consultation with a board-certified physician from CirrusMD, a contracted third-party telehealth provider that independently determined whether a CGM prescription was clinically appropriate for an individual. CirrusMD physicians evaluated members and prescribed 14 Abbott FSL 14-Day CGM sensors as part of the integrated solution, sent directly to members' homes, to cover 6 months of continuous wear while participating in the digital DSMES program. During the follow-up assessment periods (3-month and 6-month timepoints), participants in the intervention condition were instructed to wear their FSL Pro sensors and FSL 14-Day sensor at the same time. Unlike the FSL Pro sensors used for measurement of CGM-derived glycemic outcomes, members and their care team at Omada had full access to the CGM data collected via the FSL 14-Day sensors for 6 months.
Usual Care Control Condition
Participants randomized to usual care were advised during the informed consent process to continue the diabetes treatment plan as recommended by their healthcare provider from screening to the end of the study period. Usual care included oral medications, basal insulin, or non-pharmacologic therapies such as diet and exercise, in addition to ongoing medical monitoring by their healthcare provider. During the follow-up assessment periods (3-month and 6-month timepoints), participants in the usual care control condition only needed to wear their FSL Pro sensors and did not receive FSL 14-Day sensors.
Follow-Up Visits
Interim and post-study follow-up visits occurred 3 months (±14 days) and 6 months (±14 days) after randomization for all study participants, respectively. During these follow-up visits at participants' homes, contracted clinicians performed blood draws, took anthropometric measurements, assessed resting blood pressure, and reviewed cardiometabolic medication regimens. At each follow-up visit, clinicians applied and activated two FSL Pro sensors on participants for measurement and data quality purposes. Study participants were then instructed to wear the sensors for up to 14 consecutive days while blinded to the data and mail them back to the study team using a prepaid shipping envelope.
Return follow-up visits occurred outside of the +14 day range if a repeat blood draw was needed. This transpired if there was no usable data from the sample or FSL Pro sensors at no fault of the participant (e.g., mail delay causing the blood sample to be outside the testing window, sensor malfunction). Participants received compensation for all completed study visits.
"

4b-i) Report if outcomes were (self-)assessed through online questionnaires

Clearly report if outcomes were (self-)assessed through online questionnaires (as common in web-based trials) or otherwise.

	1	2	3	4	5	
subitem not at all important	○	○	○	○	○	essential

Does your paper address subitem 4b-i? *

Copy and paste relevant sections from the manuscript (include quotes in quotation marks "like this" to indicate direct quotes from your manuscript), or elaborate on this item by providing additional information not in the ms, or briefly explain why the item is not applicable/relevant for your study

The demographics, medical/diagnostic history and medication regimen were collected during the online screen of participants.

4b-ii) Report how institutional affiliations are displayed

Report how institutional affiliations are displayed to potential participants [on ehealth media], as affiliations with prestigious hospitals or universities may affect volunteer rates, use, and reactions with regards to an intervention.(Not a required item – describe only if this may bias results)

	1	2	3	4	5	
subitem not at all important	○	○	○	○	○	essential

Does your paper address subitem 4b-ii?

Copy and paste relevant sections from the manuscript (include quotes in quotation marks "like this" to indicate direct quotes from your manuscript), or elaborate on this item by providing additional information not in the ms, or briefly explain why the item is not applicable/relevant for your study

Your answer

5) The interventions for each group with sufficient details to allow replication, including how and when they were actually administered

5-i) Mention names, credential, affiliations of the developers, sponsors, and owners

Mention names, credential, affiliations of the developers, sponsors, and owners [6] (if authors/evaluators are owners or developer of the software, this needs to be declared in a “Conflict of interest” section or mentioned elsewhere in the manuscript).

	1	2	3	4	5	
subitem not at all important	○	○	○	○	○	essential

Does your paper address subitem 5-i?

Copy and paste relevant sections from the manuscript (include quotes in quotation marks "like this" to indicate direct quotes from your manuscript), or elaborate on this item by providing additional information not in the ms, or briefly explain why the item is not applicable/relevant for your study

Your answer

5-ii) Describe the history/development process

Describe the history/development process of the application and previous formative evaluations (e.g., focus groups, usability testing), as these will have an impact on adoption/use rates and help with interpreting results.

	1	2	3	4	5	
subitem not at all important	○	○	○	○	○	essential

Does your paper address subitem 5-ii?

Copy and paste relevant sections from the manuscript (include quotes in quotation marks "like this" to indicate direct quotes from your manuscript), or elaborate on this item by providing additional information not in the ms, or briefly explain why the item is not applicable/relevant for your study

Your answer

5-iii) Revisions and updating

Revisions and updating. Clearly mention the date and/or version number of the application/intervention (and comparator, if applicable) evaluated, or describe whether the intervention underwent major changes during the evaluation process, or whether the development and/or content was “frozen” during the trial. Describe dynamic components such as news feeds or changing content which may have an impact on the replicability of the intervention (for unexpected events see item 3b).

	1	2	3	4	5	
subitem not at all important	○	○	○	○	○	essential

Does your paper address subitem 5-iii?

Copy and paste relevant sections from the manuscript (include quotes in quotation marks "like this" to indicate direct quotes from your manuscript), or elaborate on this item by providing additional information not in the ms, or briefly explain why the item is not applicable/relevant for your study

Your answer

5-iv) Quality assurance methods

Provide information on quality assurance methods to ensure accuracy and quality of information provided [1], if applicable.

	1	2	3	4	5	
subitem not at all important	○	○	○	○	○	essential

Does your paper address subitem 5-iv?

Copy and paste relevant sections from the manuscript (include quotes in quotation marks "like this" to indicate direct quotes from your manuscript), or elaborate on this item by providing additional information not in the ms, or briefly explain why the item is not applicable/relevant for your study

Your answer

5-v) Ensure replicability by publishing the source code, and/or providing screenshots/screen-capture video, and/or providing flowcharts of the algorithms used

Ensure replicability by publishing the source code, and/or providing screenshots/screen-capture video, and/or providing flowcharts of the algorithms used. Replicability (i.e., other researchers should in principle be able to replicate the study) is a hallmark of scientific reporting.

	1	2	3	4	5	
subitem not at all important	○	○	○	○	○	essential

Does your paper address subitem 5-v?

Copy and paste relevant sections from the manuscript (include quotes in quotation marks "like this" to indicate direct quotes from your manuscript), or elaborate on this item by providing additional information not in the ms, or briefly explain why the item is not applicable/relevant for your study

Your answer

5-vi) Digital preservation

Digital preservation: Provide the URL of the application, but as the intervention is likely to change or disappear over the course of the years; also make sure the intervention is archived (Internet Archive, webcitation.org, and/or publishing the source code or screenshots/videos alongside the article). As pages behind login screens cannot be archived, consider creating demo pages which are accessible without login.

	1	2	3	4	5	
subitem not at all important	○	○	○	○	○	essential

Does your paper address subitem 5-vi?

Copy and paste relevant sections from the manuscript (include quotes in quotation marks "like this" to indicate direct quotes from your manuscript), or elaborate on this item by providing additional information not in the ms, or briefly explain why the item is not applicable/relevant for your study

Your answer

5-vii) Access

Access: Describe how participants accessed the application, in what setting/context, if they had to pay (or were paid) or not, whether they had to be a member of specific group. If known, describe how participants obtained "access to the platform and Internet" [1]. To ensure access for editors/reviewers/readers, consider to provide a "backdoor" login account or demo mode for reviewers/readers to explore the application (also important for archiving purposes, see vi).

	1	2	3	4	5	
subitem not at all important	○	○	○	○	○	essential

Does your paper address subitem 5-vii? *

Copy and paste relevant sections from the manuscript (include quotes in quotation marks "like this" to indicate direct quotes from your manuscript), or elaborate on this item by providing additional information not in the ms, or briefly explain why the item is not applicable/relevant for your study

"Participants randomized to the digital DSMES + CGM intervention arm completed an application for the Omada for Diabetes program, a digitally-delivered DSMES program that has achieved accreditation from the National Committee for Quality Assurance's Population Health Program and Accreditation and the Association of Diabetes Care and Education Specialists. The program pairs asynchronous health coaching from a care team comprised of certified Diabetes Prevention Program Lifestyle Coaches and Certified Diabetes Care and Education Specialists (CDCESs) with a virtual platform accessed via website or mobile app available on web-enabled devices. Upon enrollment, members receive cellularly-connected FDA devices, such as body weight scales and CGMs, along with tailored diabetes self-management and lifestyle modification support under the guidance of their care team. The care team interacts with members asynchronously through messaging on the virtual platform, regularly monitoring participants' engagement and biometric data collected during the program and using it to provide tailored feedback on diabetes self-management through proactive outreach and/or responding to member questions. Additional program features include weekly lessons with nutrition and lifestyle content, support around personalized goal setting, and online peer communities.26
After submitting an application to join the digital DSMES program as research members, digital DSMES + CGM participants completed their account setup through a one-time consultation with a board-certified physician from CirrusMD, a contracted third-party telehealth provider that independently determined whether a CGM prescription was clinically appropriate for an individual. CirrusMD physicians evaluated members and prescribed 14 Abbott FSL 14-Day CGM sensors as part of the integrated solution, sent directly to members' homes, to cover 6 months of continuous wear while participating in the digital DSMES program. During the follow-up assessment periods (3-month and 6-month timepoints), participants in the intervention condition were instructed to wear their FSL Pro sensors and FSL 14-Day sensor at the same time. Unlike the FSL Pro sensors used for measurement of CGM-derived glycemic outcomes, members and their care team at Omada had full access to the CGM data collected via the FSL 14-Day sensors for 6 months.
"

5-viii) Mode of delivery, features/functionalities/components of the intervention and comparator, and the theoretical framework

Describe mode of delivery, features/functionalities/components of the intervention and comparator, and the theoretical framework [6] used to design them (instructional strategy [1], behaviour change techniques, persuasive features, etc., see e.g., [7, 8] for terminology). This includes an in-depth description of the content (including where it is coming from and who developed it) [1]," whether [and how] it is tailored to individual circumstances and allows users to track their progress and receive feedback" [6]. This also includes a description of communication delivery channels and – if computer-mediated communication is a component – whether communication was synchronous or asynchronous [6]. It also includes information on presentation strategies [1], including page design principles, average amount of text on pages, presence of hyperlinks to other resources, etc. [1].

	1	2	3	4	5	
subitem not at all important	○	○	○	○	○	essential

Does your paper address subitem 5-viii? *

Copy and paste relevant sections from the manuscript (include quotes in quotation marks "like this" to indicate direct quotes from your manuscript), or elaborate on this item by providing additional information not in the ms, or briefly explain why the item is not applicable/relevant for your study

"CGM blood glucose values collected via FSL Pro sensors.
Digital DSMES + CGM Integrated Solution
Participants randomized to the digital DSMES + CGM intervention arm completed an application for the Omada for Diabetes program, a digitally-delivered DSMES program that has achieved accreditation from the National Committee for Quality Assurance's Population Health Program and Accreditation and the Association of Diabetes Care and Education Specialists. The program pairs asynchronous health coaching from a care team comprised of certified Diabetes Prevention Program Lifestyle Coaches and Certified Diabetes Care and Education Specialists (CDCESs) with a virtual platform accessed via website or mobile app available on web-enabled devices. Upon enrollment, members receive cellularly-connected FDA devices, such as body weight scales and CGMs, along with tailored diabetes self-management and lifestyle modification support under the guidance of their care team. The care team interacts with members asynchronously through messaging on the virtual platform, regularly monitoring participants' engagement and biometric data collected during the program and using it to provide tailored feedback on diabetes self-management through proactive outreach and/or responding to member questions. Additional program features include weekly lessons with nutrition and lifestyle content, support around personalized goal setting, and online peer communities.26
After submitting an application to join the digital DSMES program as research members, digital DSMES + CGM participants completed their account setup through a one-time consultation with a board-certified physician from CirrusMD, a contracted third-party telehealth provider that independently determined whether a CGM prescription was clinically appropriate for an individual. CirrusMD physicians evaluated members and prescribed 14 Abbott FSL 14-Day CGM sensors as part of the integrated solution, sent directly to members' homes, to cover 6 months of continuous wear while participating in the digital DSMES program. During the follow-up assessment periods (3-month and 6-month timepoints), participants in the intervention condition were instructed to wear their FSL Pro sensors and FSL 14-Day sensor at the same time. Unlike the FSL Pro sensors used for measurement of CGM-derived glycemic outcomes, members and their care team at Omada had full access to the CGM data collected via the FSL 14-Day sensors for 6 months.
Usual Care Control Condition
Participants randomized to usual care were advised during the informed consent process to continue the diabetes treatment plan as recommended by their healthcare provider from screening to the end of the study period. Usual care included oral medications, basal insulin, or non-pharmacologic therapies such as diet and exercise, in addition to ongoing medical monitoring by their healthcare provider. During the follow-up assessment periods (3-month and 6-month timepoints), participants in the usual care control condition only needed to wear their FSL Pro sensors and did not receive FSL 14-Day sensors.
"

5-ix) Describe use parameters

Describe use parameters (e.g., intended “doses” and optimal timing for use). Clarify what instructions or recommendations were given to the user, e.g., regarding timing, frequency, heaviness of use, if any, or was the intervention used ad libitum.

	1	2	3	4	5	
subitem not at all important	○	○	○	○	○	essential

Does your paper address subitem 5-ix?

Copy and paste relevant sections from the manuscript (include quotes in quotation marks "like this" to indicate direct quotes from your manuscript), or elaborate on this item by providing additional information not in the ms, or briefly explain why the item is not applicable/relevant for your study

Your answer

5-x) Clarify the level of human involvement

Clarify the level of human involvement (care providers or health professionals, also technical assistance) in the e-intervention or as co-intervention (detail number and expertise of professionals involved, if any, as well as “type of assistance offered, the timing and frequency of the support, how it is initiated, and the medium by which the assistance is delivered”. It may be necessary to distinguish between the level of human involvement required for the trial, and the level of human involvement required for a routine application outside of a RCT setting (discuss under item 21 – generalizability).

	1	2	3	4	5	
subitem not at all important	○	○	○	○	○	essential

Does your paper address subitem 5-x?

Copy and paste relevant sections from the manuscript (include quotes in quotation marks "like this" to indicate direct quotes from your manuscript), or elaborate on this item by providing additional information not in the ms, or briefly explain why the item is not applicable/relevant for your study

“The program pairs asynchronous health coaching from a care team comprised of certified Diabetes Prevention Program Lifestyle Coaches and Certified Diabetes Care and Education Specialists (CDCESs) with a virtual platform accessed via website or mobile app available on web-enabled devices. Upon enrollment, members receive cellularly-connected FDA devices, such as body weight scales and CGMs, along with tailored diabetes self-management and lifestyle modification support under the guidance of their care team. The care team interacts with members asynchronously through messaging on the virtual platform, regularly monitoring participants’ engagement and biometric data collected during the program and using it to provide tailored feedback on diabetes self-management through proactive outreach and/or responding to member questions.”

5-xi) Report any prompts/reminders used

Report any prompts/reminders used: Clarify if there were prompts (letters, emails, phone calls, SMS) to use the application, what triggered them, frequency etc. It may be necessary to distinguish between the level of prompts/reminders required for the trial, and the level of prompts/reminders for a routine application outside of a RCT setting (discuss under item 21 – generalizability).

	1	2	3	4	5	
subitem not at all important	○	○	○	○	○	essential

Does your paper address subitem 5-xi? *

Copy and paste relevant sections from the manuscript (include quotes in quotation marks "like this" to indicate direct quotes from your manuscript), or elaborate on this item by providing additional information not in the ms, or briefly explain why the item is not applicable/relevant for your study

Prompts and reminders were not used as part of the intervention in the RCT.

5-xii) Describe any co-interventions (incl. training/support)

Describe any co-interventions (incl. training/support): Clearly state any interventions that are provided in addition to the targeted eHealth intervention, as ehealth intervention may not be designed as stand-alone intervention. This includes training sessions and support [1]. It may be necessary to distinguish between the level of training required for the trial, and the level of training for a routine application outside of a RCT setting (discuss under item 21 – generalizability.

	1	2	3	4	5	
subitem not at all important	○	○	○	○	○	essential

Does your paper address subitem 5-xii? *

Copy and paste relevant sections from the manuscript (include quotes in quotation marks "like this" to indicate direct quotes from your manuscript), or elaborate on this item by providing additional information not in the ms, or briefly explain why the item is not applicable/relevant for your study

This item is not applicable.

6a) Completely defined pre-specified primary and secondary outcome measures, including how and when they were assessed

Does your paper address CONSORT subitem 6a? *

Copy and paste relevant sections from the manuscript (include quotes in quotation marks "like this" to indicate direct quotes from your manuscript), or elaborate on this item by providing additional information not in the ms, or briefly explain why the item is not applicable/relevant for your study

"Follow-Up Visits
Interim and post-study follow-up visits occurred 3 months (±14 days) and 6 months (±14 days) after randomization for all study participants, respectively. During these follow-up visits at participants' homes, contracted clinicians performed blood draws, took anthropometric measurements, assessed resting blood pressure, and reviewed cardiometabolic medication regimens. At each follow-up visit, clinicians applied and activated two FSL Pro sensors on participants for measurement and data quality purposes. Study participants were then instructed to wear the sensors for up to 14 consecutive days while blinded to the data and mail them back to the study team using a prepaid shipping envelope.
Return follow-up visits occurred outside of the +14 day range if a repeat blood draw was needed. This transpired if there was no usable data from the sample or FSL Pro sensors at no fault of the participant (e.g., mail delay causing the blood sample to be outside the testing window, sensor malfunction). Participants received compensation for all completed study visits.
Measures
Study outcomes were compared between the digital DSMES program + CGM versus usual care at Months 3 and 6. The primary outcome was HbA1c level. Secondary outcomes measured via the FSL Pro sensors included CGM 14-day % TIR, % time above range (TAR), and % time below range (TBR). Furthermore, differences in mean blood glucose, standard deviation (SD) of blood glucose, coefficient of variation, and glucose management indicator (GMI) were examined. Lastly, body weight, resting systolic and diastolic blood pressure, and diabetes distress27 were examined. Engagement in the digital DSMES + CGM integrated solution was measured via five metrics: average number of meals tracked per week using the digital DSMES app, average number of physical activity bouts tracked per week (manually self-reported in the digital DSMES app or synced from a wearable device), average number of weigh-ins per week using the provided cellularly-connected body weight scale, average number of messages sent to their care team per week, and median number of days in which participants had at least one CGM reading from the FSL 14-Day device (connected through the digital DSMES app)."

6a-i) Online questionnaires: describe if they were validated for online use and apply CHERRIES items to describe how the questionnaires were designed/deployed

If outcomes were obtained through online questionnaires, describe if they were validated for online use and apply CHERRIES items to describe how the questionnaires were designed/deployed [9].

	1	2	3	4	5	
subitem not at all important	○	○	○	○	○	essential

Does your paper address subitem 6a-i?

Copy and paste relevant sections from manuscript text

Your answer

6a-ii) Describe whether and how “use” (including intensity of use/dosage) was defined/measured/monitored

Describe whether and how “use” (including intensity of use/dosage) was defined/measured/monitored (logins, logfile analysis, etc.). Use/adoption metrics are important process outcomes that should be reported in any ehealth trial.

	1	2	3	4	5	
subitem not at all important	○	○	○	○	○	essential

Does your paper address subitem 6a-ii?

Copy and paste relevant sections from manuscript text

“Engagement in the digital DSMES + CGM integrated solution was measured via five metrics: average number of meals tracked per week using the digital DSMES app, average number of physical activity bouts tracked per week (manually self-reported in the digital DSMES app or synced from a wearable device), average number of weigh-ins per week using the provided cellularly-connected body weight scale, average number of messages sent to their care team per week, and median number of days in which participants had at least one CGM reading from the FSL 14-Day device (connected through the digital DSMES app).”

6a-iii) Describe whether, how, and when qualitative feedback from participants was obtained

Describe whether, how, and when qualitative feedback from participants was obtained (e.g., through emails, feedback forms, interviews, focus groups).

	1	2	3	4	5	
subitem not at all important	○	○	○	○	○	essential

Does your paper address subitem 6a-iii?

Copy and paste relevant sections from manuscript text

Your answer

6b) Any changes to trial outcomes after the trial commenced, with reasons

Does your paper address CONSORT subitem 6b? *

Copy and paste relevant sections from the manuscript (include quotes in quotation marks "like this" to indicate direct quotes from your manuscript), or elaborate on this item by providing additional information not in the ms, or briefly explain why the item is not applicable/relevant for your study

This item is not applicable.

7a) How sample size was determined

NPT: When applicable, details of whether and how the clustering by care provides or centers was addressed

7a-i) Describe whether and how expected attrition was taken into account when calculating the sample size

Describe whether and how expected attrition was taken into account when calculating the sample size.

	1	2	3	4	5	
subitem not at all important	○	○	○	○	○	essential

Does your paper address subitem 7a-i?

Copy and paste relevant sections from manuscript title (include quotes in quotation marks "like this" to indicate direct quotes from your manuscript), or elaborate on this item by providing additional information not in the ms, or briefly explain why the item is not applicable/relevant for your study

Your answer

7b) When applicable, explanation of any interim analyses and stopping guidelines

Does your paper address CONSORT subitem 7b? *

Copy and paste relevant sections from the manuscript (include quotes in quotation marks "like this" to indicate direct quotes from your manuscript), or elaborate on this item by providing additional information not in the ms, or briefly explain why the item is not applicable/relevant for your study

This item is not applicable.

8a) Method used to generate the random allocation sequence
NPT: When applicable, how care providers were allocated to each trial group

Does your paper address CONSORT subitem 8a? *

Copy and paste relevant sections from the manuscript (include quotes in quotation marks "like this" to indicate direct quotes from your manuscript), or elaborate on this item by providing additional information not in the ms, or briefly explain why the item is not applicable/relevant for your study

"Each eligible participant was randomized to one of the two conditions (digital DSMES + CGM or usual care) according to an Evidation-controlled algorithm that used randomly permuted blocks of fixed size. Randomization was stratified by gender to ensure nearly equal sample sizes for the two treatment arms for each gender.
The assessment staff members (i.e., clinicians conducting home visits, Quest Diagnostics) and the Evidation-designated statistician that developed the statistical analysis plan were blinded to the intervention assignment during the study period. Adverse-event adjudicators, designated research personnel at Evidation and Omada, as well as Omada coaching staff, were not blinded. Participants were not blinded to their assignment, but they were blinded to their HbA1c levels and CGM blood glucose values collected via FSL Pro sensors."

8b) Type of randomisation; details of any restriction (such as blocking and block size)

Does your paper address CONSORT subitem 8b? *

Copy and paste relevant sections from the manuscript (include quotes in quotation marks "like this" to indicate direct quotes from your manuscript), or elaborate on this item by providing additional information not in the ms, or briefly explain why the item is not applicable/relevant for your study

"Each eligible participant was randomized to one of the two conditions (digital DSMES + CGM or usual care) according to an Evidation-controlled algorithm that used randomly permuted blocks of fixed size. Randomization was stratified by gender to ensure nearly equal sample sizes for the two treatment arms for each gender.
The assessment staff members (i.e., clinicians conducting home visits, Quest Diagnostics) and the Evidation-designated statistician that developed the statistical analysis plan were blinded to the intervention assignment during the study period. Adverse-event adjudicators, designated research personnel at Evidation and Omada, as well as Omada coaching staff, were not blinded. Participants were not blinded to their assignment, but they were blinded to their HbA1c levels and CGM blood glucose values collected via FSL Pro sensors."

9) Mechanism used to implement the random allocation sequence (such as sequentially numbered containers), describing any steps taken to conceal the sequence until interventions were assigned

Does your paper address CONSORT subitem 9? *

Copy and paste relevant sections from the manuscript (include quotes in quotation marks "like this" to indicate direct quotes from your manuscript), or elaborate on this item by providing additional information not in the ms, or briefly explain why the item is not applicable/relevant for your study

"Each eligible participant was randomized to one of the two conditions (digital DSMES + CGM or usual care) according to an Evidation-controlled algorithm that used randomly permuted blocks of fixed size. Randomization was stratified by gender to ensure nearly equal sample sizes for the two treatment arms for each gender.
The assessment staff members (i.e., clinicians conducting home visits, Quest Diagnostics) and the Evidation-designated statistician that developed the statistical analysis plan were blinded to the intervention assignment during the study period. Adverse-event adjudicators, designated research personnel at Evidation and Omada, as well as Omada coaching staff, were not blinded. Participants were not blinded to their assignment, but they were blinded to their HbA1c levels and CGM blood glucose values collected via FSL Pro sensors."

10) Who generated the random allocation sequence, who enrolled participants, and who assigned participants to interventions

Does your paper address CONSORT subitem 10? *

Copy and paste relevant sections from the manuscript (include quotes in quotation marks "like this" to indicate direct quotes from your manuscript), or elaborate on this item by providing additional information not in the ms, or briefly explain why the item is not applicable/relevant for your study

"Each eligible participant was randomized to one of the two conditions (digital DSMES + CGM or usual care) according to an Evidation-controlled algorithm that used randomly permuted blocks of fixed size. Randomization was stratified by gender to ensure nearly equal sample sizes for the two treatment arms for each gender.
The assessment staff members (i.e., clinicians conducting home visits, Quest Diagnostics) and the Evidation-designated statistician that developed the statistical analysis plan were blinded to the intervention assignment during the study period. Adverse-event adjudicators, designated research personnel at Evidation and Omada, as well as Omada coaching staff, were not blinded. Participants were not blinded to their assignment, but they were blinded to their HbA1c levels and CGM blood glucose values collected via FSL Pro sensors."

11a) If done, who was blinded after assignment to interventions (for example, participants, care providers, those assessing outcomes) and how

NPT: Whether or not administering co-interventions were blinded to group assignment

11a-i) Specify who was blinded, and who wasn’t

Specify who was blinded, and who wasn’t. Usually, in web-based trials it is not possible to blind the participants [1, 3] (this should be clearly acknowledged), but it may be possible to blind outcome assessors, those doing data analysis or those administering co-interventions (if any).

	1	2	3	4	5	
subitem not at all important	○	○	○	○	○	essential

Does your paper address subitem 11a-i? *

Copy and paste relevant sections from the manuscript (include quotes in quotation marks "like this" to indicate direct quotes from your manuscript), or elaborate on this item by providing additional information not in the ms, or briefly explain why the item is not applicable/relevant for your study

"During the home visits, they collected up to 4 mL of blood via venipuncture, anthropometric measurements, resting blood pressure, and a list of participants' cardiometabolic medications. They also applied and activated two FSL Pro CGM sensors on the participants for measurement purposes, and participants were instructed to wear the FSL Pro CGM sensors for up to 14 days while blinded to the data. Afterwards, participants mailed the devices to the study team for data download and TIR analysis. "
"Each eligible participant was randomized to one of the two conditions (digital DSMES + CGM or usual care) according to an Evidation-controlled algorithm that used randomly permuted blocks of fixed size. Randomization was stratified by gender to ensure nearly equal sample sizes for the two treatment arms for each gender.
The assessment staff members (i.e., clinicians conducting home visits, Quest Diagnostics) and the Evidation-designated statistician that developed the statistical analysis plan were blinded to the intervention assignment during the study period. Adverse-event adjudicators, designated research personnel at Evidation and Omada, as well as Omada coaching staff, were not blinded. Participants were not blinded to their assignment, but they were blinded to their HbA1c levels and CGM blood glucose values collected via FSL Pro sensors."

11a-ii) Discuss e.g., whether participants knew which intervention was the “intervention of interest” and which one was the “comparator”

Informed consent procedures (4a-ii) can create biases and certain expectations - discuss e.g., whether participants knew which intervention was the “intervention of interest” and which one was the “comparator”.

	1	2	3	4	5	
subitem not at all important	○	○	○	○	○	essential

Does your paper address subitem 11a-ii?

Copy and paste relevant sections from the manuscript (include quotes in quotation marks "like this" to indicate direct quotes from your manuscript), or elaborate on this item by providing additional information not in the ms, or briefly explain why the item is not applicable/relevant for your study

Yes, participants knew they would be randomized to the Omada program or standard of care; all control group participants had the opportunity to sign up for the Omada program at the end of the study.

11b) If relevant, description of the similarity of interventions

(this item is usually not relevant for ehealth trials as it refers to similarity of a placebo or sham intervention to a active medication/intervention)

Does your paper address CONSORT subitem 11b? *

Copy and paste relevant sections from the manuscript (include quotes in quotation marks "like this" to indicate direct quotes from your manuscript), or elaborate on this item by providing additional information not in the ms, or briefly explain why the item is not applicable/relevant for your study

This is not applicable for our study.

12a) Statistical methods used to compare groups for primary and secondary outcomes

NPT: When applicable, details of whether and how the clustering by care providers or centers was addressed

Does your paper address CONSORT subitem 12a? *

Copy and paste relevant sections from the manuscript (include quotes in quotation marks "like this" to indicate direct quotes from your manuscript), or elaborate on this item by providing additional information not in the ms, or briefly explain why the item is not applicable/relevant for your study

"Linear mixed effects models were used to analyze both the primary and secondary outcomes using both per-protocol (PP) and intention-to-treat (ITT) methodology. For PP, we excluded participants who were randomized to the intervention condition but never enrolled in the digital DSMES + CGM integrated solution (n=9). For the primary outcome model, fixed effects included timepoint, condition, and a timepoint-by-condition interaction. A random slope for time and a random intercept for participant were included. For the secondary outcome models, fixed effects included timepoint, condition, and a timepoint by condition interaction. Given that several of the models would not converge when a random slope for time was included, only a random intercept for participant was included in the models.29 All models utilized restricted maximum likelihood estimation with an unstructured covariance matrix. Estimated marginal means were obtained from the models, and planned contrasts were conducted to examine differences by condition at Months 3 and 6."

12a-i) Imputation techniques to deal with attrition / missing values

Imputation techniques to deal with attrition / missing values: Not all participants will use the intervention/comparator as intended and attrition is typically high in ehealth trials. Specify how participants who did not use the application or dropped out from the trial were treated in the statistical analysis (a complete case analysis is strongly discouraged, and simple imputation techniques such as LOCF may also be problematic [4]).

	1	2	3	4	5	
subitem not at all important	○	○	○	○	○	essential

Does your paper address subitem 12a-i? *

Copy and paste relevant sections from the manuscript (include quotes in quotation marks "like this" to indicate direct quotes from your manuscript), or elaborate on this item by providing additional information not in the ms, or briefly explain why the item is not applicable/relevant for your study

"Linear mixed effects models were used to analyze both the primary and secondary outcomes using both per-protocol (PP) and intention-to-treat (ITT) methodology. For PP, we excluded participants who were randomized to the intervention condition but never enrolled in the digital DSMES + CGM integrated solution (n=9). For the primary outcome model, fixed effects included timepoint, condition, and a timepoint-by-condition interaction. A random slope for time and a random intercept for participant were included. For the secondary outcome models, fixed effects included timepoint, condition, and a timepoint by condition interaction. Given that several of the models would not converge when a random slope for time was included, only a random intercept for participant was included in the models.29 All models utilized restricted maximum likelihood estimation with an unstructured covariance matrix. Estimated marginal means were obtained from the models, and planned contrasts were conducted to examine differences by condition at Months 3 and 6. Results from the planned comparisons are reported in Figure 1 for the primary outcome and Tables 2 and 3 for the secondary outcomes."

12b) Methods for additional analyses, such as subgroup analyses and adjusted analyses

Does your paper address CONSORT subitem 12b? *

Copy and paste relevant sections from the manuscript (include quotes in quotation marks "like this" to indicate direct quotes from your manuscript), or elaborate on this item by providing additional information not in the ms, or briefly explain why the item is not applicable/relevant for your study

"Age, BMI, sex, cardiovascular medication use, and type 2 diabetes medication use were examined as potential covariates by exploring condition differences in each covariate and examining associations between each covariate and outcome. Given that there were no condition differences on any of the potential covariates, no covariates were included in the analyses."
"Estimated marginal means were obtained from the models, and planned contrasts were conducted to examine differences by condition at Months 3 and 6"

X26) REB/IRB Approval and Ethical Considerations [recommended as subheading under "Methods"] (not a CONSORT item)

X26-i) Comment on ethics committee approval

	1	2	3	4	5	
subitem not at all important	◯	◯	◯	◯	◯	essential

Does your paper address subitem X26-i?

Copy and paste relevant sections from the manuscript (include quotes in quotation marks "like this" to indicate direct quotes from your manuscript), or elaborate on this item by providing additional information not in the ms, or briefly explain why the item is not applicable/relevant for your study

Your answer

x26-ii) Outline informed consent procedures

Outline informed consent procedures e.g., if consent was obtained offline or online (how? Checkbox, etc.?), and what information was provided (see 4a-ii). See [6] for some items to be included in informed consent documents.

	1	2	3	4	5	
subitem not at all important	◯	◯	◯	◯	◯	essential

Does your paper address subitem X26-ii?

Copy and paste relevant sections from the manuscript (include quotes in quotation marks "like this" to indicate direct quotes from your manuscript), or elaborate on this item by providing additional information not in the ms, or briefly explain why the item is not applicable/relevant for your study

Your answer

X26-iii) Safety and security procedures

Safety and security procedures, incl. privacy considerations, and any steps taken to reduce the likelihood or detection of harm (e.g., education and training, availability of a hotline)

	1	2	3	4	5	
subitem not at all important	○	○	○	○	○	essential

Does your paper address subitem X26-iii?

Copy and paste relevant sections from the manuscript (include quotes in quotation marks "like this" to indicate direct quotes from your manuscript), or elaborate on this item by providing additional information not in the ms, or briefly explain why the item is not applicable/relevant for your study

Your answer

RESULTS

13a) For each group, the numbers of participants who were randomly assigned, received intended treatment, and were analysed for the primary outcome

NPT: The number of care providers or centers performing the intervention in each group and the number of patients treated by each care provider in each center

Does your paper address CONSORT subitem 13a? *

Copy and paste relevant sections from the manuscript (include quotes in quotation marks "like this" to indicate direct quotes from your manuscript), or elaborate on this item by providing additional information not in the ms, or briefly explain why the item is not applicable/relevant for your study

"A total of 39,506 individuals were initially screened for eligibility (See Supplemental Figure S1 for CONSORT diagram and Supplemental Table S1 for CONSORT checklist). Of these, 594 were screened as eligible, and 292 individuals completed an informed consent form and the baseline survey. After participating in a secondary screening (n=227), 112 individuals were disqualified due to HbA1c or TIR eligibility criteria, and 15 individuals were withdrawn/lost to follow-up. Thus, the final sample included 100 participants randomized to the digital DSMES + CGM intervention condition (n=51) or usual care (n=49). A total of 46 digital DSMES + CGM participants and 43 usual care participants completed their 3-month visit, and 43 digital DSMES + CGM participants and 45 usual care participants completed their 6-month visit."

13b) For each group, losses and exclusions after randomisation, together with reasons

Does your paper address CONSORT subitem 13b? (NOTE: Preferably, this is shown in a CONSORT flow diagram) *

Copy and paste relevant sections from the manuscript (include quotes in quotation marks "like this" to indicate direct quotes from your manuscript), or elaborate on this item by providing additional information not in the ms, or briefly explain why the item is not applicable/relevant for your study

Yes, we explain in the CONSORT diagram why participant were withdrawn/lost to follow-up. "Participants were either withdrawn due to ineligibility criteria or were marked lost to follow-up if the research staff was unable to reach participants after multiple attempts."

13b-i) Attrition diagram

Strongly recommended: An attrition diagram (e.g., proportion of participants still logging in or using the intervention/comparator in each group plotted over time, similar to a survival curve) or other figures or tables demonstrating usage/dose/engagement.

	1	2	3	4	5	
subitem not at all important	○	○	○	○	○	essential

Does your paper address subitem 13b-i?

Copy and paste relevant sections from the manuscript or cite the figure number if applicable (include quotes in quotation marks "like this" to indicate direct quotes from your manuscript), or elaborate on this item by providing additional information not in the ms, or briefly explain why the item is not applicable/relevant for your study

Your answer

14a) Dates defining the periods of recruitment and follow-up

Does your paper address CONSORT subitem 14a? *

Copy and paste relevant sections from the manuscript (include quotes in quotation marks "like this" to indicate direct quotes from your manuscript), or elaborate on this item by providing additional information not in the ms, or briefly explain why the item is not applicable/relevant for your study

"During the initial recruitment period (June-October 2022), participants were also required to have a percent time in range (TIR) between 70-180 mg/dL of <60% prior to enrollment, which was eventually removed from the eligibility criteria to reduce participant burden and improve study activity workflow during the remainder of the recruitment period (October 2022-May 2023). "
"Interim and post-study follow-up visits occurred 3 months (±14 days) and 6 months (±14 days) after randomization for all study participants, respectively."

14a-i) Indicate if critical “secular events” fell into the study period

Indicate if critical “secular events” fell into the study period, e.g., significant changes in Internet resources available or “changes in computer hardware or Internet delivery resources”

	1	2	3	4	5	
subitem not at all important	○	○	○	○	○	essential

Does your paper address subitem 14a-i?

Copy and paste relevant sections from the manuscript (include quotes in quotation marks "like this" to indicate direct quotes from your manuscript), or elaborate on this item by providing additional information not in the ms, or briefly explain why the item is not applicable/relevant for your study

Your answer

14b) Why the trial ended or was stopped (early)

Does your paper address CONSORT subitem 14b? *

Copy and paste relevant sections from the manuscript (include quotes in quotation marks "like this" to indicate direct quotes from your manuscript), or elaborate on this item by providing additional information not in the ms, or briefly explain why the item is not applicable/relevant for your study

This study was not stopped early so this is not applicable.

15) A table showing baseline demographic and clinical characteristics for each group

NPT: When applicable, a description of care providers (case volume, qualification, expertise, etc.) and centers (volume) in each group

Does your paper address CONSORT subitem 15? *

Copy and paste relevant sections from the manuscript (include quotes in quotation marks "like this" to indicate direct quotes from your manuscript), or elaborate on this item by providing additional information not in the ms, or briefly explain why the item is not applicable/relevant for your study

"Baseline demographic and clinical characteristics of the study population are presented in Table 1. "

15-i) Report demographics associated with digital divide issues

In ehealth trials it is particularly important to report demographics associated with digital divide issues, such as age, education, gender, social-economic status, computer/Internet/ehealth literacy of the participants, if known.

	1	2	3	4	5	
subitem not at all important	○	○	○	○	○	essential

Does your paper address subitem 15-i? *

Copy and paste relevant sections from the manuscript (include quotes in quotation marks "like this" to indicate direct quotes from your manuscript), or elaborate on this item by providing additional information not in the ms, or briefly explain why the item is not applicable/relevant for your study

"Baseline demographic and clinical characteristics of the study population are presented in Table 1."

16) For each group, number of participants (denominator) included in each analysis and whether the analysis was by original assigned groups

16-i) Report multiple “denominators” and provide definitions

Report multiple “denominators” and provide definitions: Report N’s (and effect sizes) “across a range of study participation [and use] thresholds” [1], e.g., N exposed, N consented, N used more than x times, N used more than y weeks, N participants “used” the intervention/comparator at specific pre-defined time points of interest (in absolute and relative numbers per group). Always clearly define “use” of the intervention.

	1	2	3	4	5	
subitem not at all important	○	○	○	○	○	essential

Does your paper address subitem 16-i? *

Copy and paste relevant sections from the manuscript (include quotes in quotation marks "like this" to indicate direct quotes from your manuscript), or elaborate on this item by providing additional information not in the ms, or briefly explain why the item is not applicable/relevant for your study

"Thus, the final sample included 100 participants randomized to the digital DSMES + CGM intervention condition (n=51) or usual care (n=49). A total of 46 digital DSMES + CGM participants and 43 usual care participants completed their 3-month visit, and 43 digital DSMES + CGM participants and 45 usual care participants completed their 6-month visit."

16-ii) Primary analysis should be intent-to-treat

Primary analysis should be intent-to-treat, secondary analyses could include comparing only “users”, with the appropriate caveats that this is no longer a randomized sample (see 18-i).

	1	2	3	4	5	
subitem not at all important	○	○	○	○	○	essential

Does your paper address subitem 16-ii?

Copy and paste relevant sections from the manuscript (include quotes in quotation marks "like this" to indicate direct quotes from your manuscript), or elaborate on this item by providing additional information not in the ms, or briefly explain why the item is not applicable/relevant for your study

“Linear mixed effects models were used to analyze both the primary and secondary outcomes using both per-protocol (PP) and intention-to-treat (ITT) methodology.”
“In the ITT analysis, participants in the digital DSMES + CGM condition had significantly lower HbA1c than usual care at Month 3 (-0.7% [95% CI -1.4 to -0.1] or -8.1 mmol/mol [95% CI -15.5 to -0.7], P=.03) and clinically meaningful reductions in HbA1c at Month 6 (-0.6% [95% CI -1.4 to 0.2] or -6.9 mmol/mol [95% CI -15.7 to 1.9], P=.12).”
“CGM-derived glycemic measures were collected using the blinded FSL Pro sensors. In the ITT analyses for Month 3, participants in the digital DSMES + CGM condition had a 13.5% (95% CI 0.3 to 26.6; P=.045) higher TIR and a 16.9% (95% CI -30.4 to -3.3; P=.02) lower TAR >250 mg/dL than participants in usual care. In addition, at Month 3, participants in the digital DSMES + CGM condition had 39.8 mg/dL (95% CI -72.2 to -7.4; P=.02) lower mean glucose and 0.9% (95% CI -1.7 to -0.2; P=.02) lower GMI than participants in usual care.”
“At Month 6, participants in the digital DSMES + CGM condition had a 14.6% (95% CI 1.0 to 28.2; P=.04) higher TIR, a 14.9% (95% CI -29.0 to -0.9; P=.04) lower TAR >180 mg/dL, and a 17.9% (95% CI -32.0 to -3.8; P=.01) lower TAR >250 mg/dL than participants in usual care. In addition, at Month 6, participants in the digital DSMES + CGM condition had 36.4 mg/dL (95% CI -70.0 to -2.9; P=.03) lower mean glucose, 5.2 mg/dL (95% CI -10.3 to -0.01; P=.047) lower SD of glucose, and 0.9% (95% CI -1.7 to -0.1; P=.03) lower GMI than participants in usual care.”
“Finally, no statistically significant differences between the intervention and control groups were seen for weight, systolic or diastolic blood pressure, or diabetes distress at Month 3 or Month 6.”

17a) For each primary and secondary outcome, results for each group, and the estimated effect size and its precision (such as 95% confidence interval)

Does your paper address CONSORT subitem 17a? *

Copy and paste relevant sections from the manuscript (include quotes in quotation marks "like this" to indicate direct quotes from your manuscript), or elaborate on this item by providing additional information not in the ms, or briefly explain why the item is not applicable/relevant for your study

"In the ITT analysis, participants in the digital DSMES + CGM condition had significantly lower HbA1c than usual care at Month 3 (-0.7% [95% CI -1.4 to -0.1] or -8.1 mmol/mol [95% CI -15.5 to -0.7], P=.03) and clinically meaningful reductions in HbA1c at Month 6 (-0.6% [95% CI -1.4 to 0.2] or -6.9 mmol/mol [95% CI -15.7 to 1.9], P=.12).
In the PP analysis (presented in Figure 1), participants in the digital DSMES + CGM condition had significantly lower HbA1c than usual care at both Month 3 (-1.0% [95% CI -1.6 to -0.3] or -10.5 mmol/mol [95% CI -17.9 to -3.1], P=.006) and Month 6 (-0.8% [95% CI -1.6 to -0.02] or -9.1 mmol/mol [95% CI -18.0 to -0.2], P=.046)."
"CGM-derived glycemic measures were collected using the blinded FSL Pro sensors. In the ITT analyses for Month 3, participants in the digital DSMES + CGM condition had a 13.5% (95% CI 0.3 to 26.6; P=.045) higher TIR and a 16.9% (95% CI -30.4 to -3.3; P=.02) lower TAR >250 mg/dL than participants in usual care. In addition, at Month 3, participants in the digital DSMES + CGM condition had 39.8 mg/dL (95% CI -72.2 to -7.4; P=.02) lower mean glucose and 0.9% (95% CI -1.7 to -0.2; P=.02) lower GMI than participants in usual care.
At Month 6, participants in the digital DSMES + CGM condition had a 14.6% (95% CI 1.0 to 28.2; P=.04) higher TIR, a 14.9% (95% CI -29.0 to -0.9; P=.04) lower TAR >180 mg/dL, and a 17.9% (95% CI -32.0 to -3.8; P=.01) lower TAR >250 mg/dL than participants in usual care. In addition, at Month 6, participants in the digital DSMES + CGM condition had 36.4 mg/dL (95% CI -70.0 to -2.9; P=.03) lower mean glucose, 5.2 mg/dL (95% CI -10.3 to -0.01; P=.047) lower SD of glucose, and 0.9% (95% CI -1.7 to -0.1; P=.03) lower GMI than participants in usual care.
Finally, no statistically significant differences between the intervention and control groups were seen for weight, systolic or diastolic blood pressure, or diabetes distress at Month 3 or Month 6.
Results for the PP analyses are presented in Table 2 for Month 3 outcomes, Table 3 for Month 6 outcomes, and Figure 2. These results remained largely the same as the ITT analyses with two exceptions: participants in the digital DSMES + CGM condition had significantly lower TAR >180 mg/dL (-16.3% [95% CI -30.3 to -2.3]; P=.02) and SD of glucose (-5.8 mg/dL [95% CI -11.0 to -0.7]; P=.03) than usual care at Month 3."

17a-i) Presentation of process outcomes such as metrics of use and intensity of use

In addition to primary/secondary (clinical) outcomes, the presentation of process outcomes such as metrics of use and intensity of use (dose, exposure) and their operational definitions is critical. This does not only refer to metrics of attrition (13-b) (often a binary variable), but also to more continuous exposure metrics such as “average session length”. These must be accompanied by a technical description how a metric like a “session” is defined (e.g., timeout after idle time) [1] (report under item 6a).

	1	2	3	4	5	
subitem not at all important	○	○	○	○	○	essential

Does your paper address subitem 17a-i?

Copy and paste relevant sections from the manuscript (include quotes in quotation marks "like this" to indicate direct quotes from your manuscript), or elaborate on this item by providing additional information not in the ms, or briefly explain why the item is not applicable/relevant for your study

“Of those who enrolled in the digital DSMES + CGM integrated solution (n=42), participants tracked an average of 4.6 meals (SD=6.7) and 4.3 physical activity bouts (SD=3.1) per week within the digital DSMES app. Participants also weighed themselves an average of 3.3 times (SD=2.5) per week using the cellularly-connected body weight scale as part of the digital DSMES program. Participants sent an average of 1.2 messages (SD=1.1) to their care team per week. The median number of days per week in which participants had at least one CGM reading from the FSL 14-Day sensor was 4.8.”

17b) For binary outcomes, presentation of both absolute and relative effect sizes is recommended

Does your paper address CONSORT subitem 17b? *

Copy and paste relevant sections from the manuscript (include quotes in quotation marks "like this" to indicate direct quotes from your manuscript), or elaborate on this item by providing additional information not in the ms, or briefly explain why the item is not applicable/relevant for your study

This is not applicable to our study.

18) Results of any other analyses performed, including subgroup analyses and adjusted analyses, distinguishing pre-specified from exploratory

Does your paper address CONSORT subitem 18? *

Copy and paste relevant sections from the manuscript (include quotes in quotation marks "like this" to indicate direct quotes from your manuscript), or elaborate on this item by providing additional information not in the ms, or briefly explain why the item is not applicable/relevant for your study

"Of members who reported taking diabetes medication at one or more of the three study visits (n = 95), 91% (n = 86) reported a change in their medication over time. Specifically, a total of 53% of members started a new medication and 58% of members stopped a medication during the study period, with 35% of members increasing the total number of medications reported and 42% decreasing the total number of medications reported during the study period. There were no significant differences in baseline characteristics (including baseline diabetes medications) or in the number of members who started or stopped a medication, used insulin, increased or decreased the number of medications reported, or had any medication change during the study period between the two conditions. When looking only at members who took oral medication only at baseline (n=67), there were no differences in baseline characteristics between the two conditions."
"Of those who enrolled in the digital DSMES + CGM integrated solution (n=42), participants tracked an average of 4.6 meals (SD=6.7) and 4.3 physical activity bouts (SD=3.1) per week within the digital DSMES app. Participants also weighed themselves an average of 3.3 times (SD=2.5) per week using the cellularly-connected body weight scale as part of the digital DSMES program. Participants sent an average of 1.2 messages (SD=1.1) to their care team per week. The median number of days per week in which participants had at least one CGM reading from the FSL 14-Day sensor was 4.8."

18-i) Subgroup analysis of comparing only users

A subgroup analysis of comparing only users is not uncommon in ehealth trials, but if done, it must be stressed that this is a self-selected sample and no longer an unbiased sample from a randomized trial (see 16-iii).

	1	2	3	4	5	
subitem not at all important	○	○	○	○	○	essential

Does your paper address subitem 18-i?

Copy and paste relevant sections from the manuscript (include quotes in quotation marks "like this" to indicate direct quotes from your manuscript), or elaborate on this item by providing additional information not in the ms, or briefly explain why the item is not applicable/relevant for your study

“In the PP analysis (presented in Figure 1), participants in the digital DSMES + CGM condition had significantly lower HbA1c than usual care at both Month 3 (-1.0% [95% CI -1.6 to -0.3] or -10.5 mmol/mol [95% CI -17.9 to -3.1], P=.006) and Month 6 (-0.8% [95% CI -1.6 to -0.02] or -9.1 mmol/mol [95% CI -18.0 to -0.2], P=.046).”
“Results for the PP analyses are presented in Table 2 for Month 3 outcomes, Table 3 for Month 6 outcomes, and Figure 2. These results remained largely the same as the ITT analyses with two exceptions: participants in the digital DSMES + CGM condition had significantly lower TAR >180 mg/dL (-16.3% [95% CI -30.3 to -2.3]; P=.02) and SD of glucose (-5.8 mg/dL [95% CI -11.0 to -0.7]; P=.03) than usual care at Month 3.”

19) All important harms or unintended effects in each group

(for specific guidance see CONSORT for harms)

Does your paper address CONSORT subitem 19? *

Copy and paste relevant sections from the manuscript (include quotes in quotation marks "like this" to indicate direct quotes from your manuscript), or elaborate on this item by providing additional information not in the ms, or briefly explain why the item is not applicable/relevant for your study

"There were no reported serious adverse events related to the study devices or program, and the number of adverse events did not differ significantly across conditions."

19-i) Include privacy breaches, technical problems

Include privacy breaches, technical problems. This does not only include physical "harm" to participants, but also incidents such as perceived or real privacy breaches [1], technical problems, and other unexpected/unintended incidents. "Unintended effects" also includes unintended positive effects [2].

	1	2	3	4	5	
subitem not at all important	○	○	○	○	○	essential

Does your paper address subitem 19-i?

Copy and paste relevant sections from the manuscript (include quotes in quotation marks "like this" to indicate direct quotes from your manuscript), or elaborate on this item by providing additional information not in the ms, or briefly explain why the item is not applicable/relevant for your study

Your answer

19-ii) Include qualitative feedback from participants or observations from staff/researchers

Include qualitative feedback from participants or observations from staff/researchers, if available, on strengths and shortcomings of the application, especially if they point to unintended/unexpected effects or uses. This includes (if available) reasons for why people did or did not use the application as intended by the developers.

	1	2	3	4	5	
subitem not at all important	○	○	○	○	○	essential

Does your paper address subitem 19-ii?

Copy and paste relevant sections from the manuscript (include quotes in quotation marks "like this" to indicate direct quotes from your manuscript), or elaborate on this item by providing additional information not in the ms, or briefly explain why the item is not applicable/relevant for your study

Your answer

DISCUSSION

22) Interpretation consistent with results, balancing benefits and harms, and considering other relevant evidence

NPT: In addition, take into account the choice of the comparator, lack of or partial blinding, and unequal expertise of care providers or centers in each group

22-i) Restate study questions and summarize the answers suggested by the data, starting with primary outcomes and process outcomes (use)

Restate study questions and summarize the answers suggested by the data, starting with primary outcomes and process outcomes (use).

	1	2	3	4	5	
subitem not at all important	○	○	○	○	○	essential

Does your paper address subitem 22-i? *

Copy and paste relevant sections from the manuscript (include quotes in quotation marks "like this" to indicate direct quotes from your manuscript), or elaborate on this item by providing additional information not in the ms, or briefly explain why the item is not applicable/relevant for your study

"Results from this RCT evaluating an integrated digital DSMES program + CGM revealed that participants randomized to the integrated solution experienced clinically meaningful and statistically significant improvements in HbA1c and CGM-derived measures of glycemic control compared to usual care over 6 months. These findings are clinically meaningful given that, on average, the intervention group saw an almost 1-point reduction in HbA1c levels over the study period–a degree of change that is twice as large as the benchmark for a clinically significant change in HbA1c of 0.5%30 and is associated with a reduced risk of diabetes-related complications, including microvascular disease, myocardial infarction, heart failure, stroke and death.31 "
"Given the focus of this trial on the integration of continuous CGM with DSMES,32 CGM-derived glycemic measures also improved in the digital DSMES + CGM condition compared to usual care. From baseline to Month 6, participants in the digital DSMES + CGM condition experienced an 18% increase in percent TIR and an 18% decrease in percent TAR >180 mg/dL, which were more favorable improvements compared to usual care, and at a magnitude of change three-fold greater than what previous research has demonstrated to be clinically meaningful.33 Additionally, participants in our integrated solution had a 1.1% lower GMI than participants in usual care at Month 6, indicating that those receiving the digital DSMES program + CGM had better glycemic control and a lower risk for diabetes-related complications.34 Together, our findings demonstrate improvement to time-in-range and reduced variability of blood glucose among participants who received the digital DSMES + CGM integrated solution compared to usual care."

22-ii) Highlight unanswered new questions, suggest future research

Highlight unanswered new questions, suggest future research.

	1	2	3	4	5	
subitem not at all important	○	○	○	○	○	essential

Does your paper address subitem 22-ii?

Copy and paste relevant sections from the manuscript (include quotes in quotation marks "like this" to indicate direct quotes from your manuscript), or elaborate on this item by providing additional information not in the ms, or briefly explain why the item is not applicable/relevant for your study

Your answer

20) Trial limitations, addressing sources of potential bias, imprecision, and, if relevant, multiplicity of analyses

20-i) Typical limitations in ehealth trials

Typical limitations in ehealth trials: Participants in ehealth trials are rarely blinded. Ehealth trials often look at a multiplicity of outcomes, increasing risk for a Type I error. Discuss biases due to non-use of the intervention/usability issues, biases through informed consent procedures, unexpected events.

	1	2	3	4	5	
subitem not at all important	○	○	○	○	○	essential

Does your paper address subitem 20-i? *

Copy and paste relevant sections from the manuscript (include quotes in quotation marks "like this" to indicate direct quotes from your manuscript), or elaborate on this item by providing additional information not in the ms, or briefly explain why the item is not applicable/relevant for your study

"We also acknowledge several limitations with the current study. First, the complexities of remote study operations, such as costs, shipping delays, scheduling conflicts, and recruitment difficulties, caused us to fall short of our intended sample size and highlighted the challenges of implementing a decentralized RCT. However, the clinically and statistically significant results observed with a smaller sample size underscore the effectiveness of the digital DSMES + CGM integrated solution. Second, nine participants randomized to the intervention did not participate in the digital DSMES program and, therefore, did not experience the integrated solution. Reasons contributing to treatment nonadherence included screen failure due to disqualifying TIR or previous exposure to the DSMES program, failure to apply to the DSMES program, and eventual loss to follow-up. That said, all nine participants had at least one follow-up assessment, so we were still able to evaluate their clinical outcomes. Finally, as part of the integrated solution, participants were expected to wear a FSL 14-Day sensor continuously for the duration of the study (along with two FSL Pro sensors at baseline, 3 months, and 6 months for measurement purposes), which may have increased participant burden and impacted adherence over the 6-month study period. However, these results likely reflect real-world compliance with CGM usage on a long-term, continuous basis."

21) Generalisability (external validity, applicability) of the trial findings

NPT: External validity of the trial findings according to the intervention, comparators, patients, and care providers or centers involved in the trial

21-i) Generalizability to other populations

Generalizability to other populations: In particular, discuss generalizability to a general Internet population, outside of a RCT setting, and general patient population, including applicability of the study results for other organizations

	1	2	3	4	5	
subitem not at all important	○	○	○	○	○	essential

Does your paper address subitem 21-i?

Copy and paste relevant sections from the manuscript (include quotes in quotation marks "like this" to indicate direct quotes from your manuscript), or elaborate on this item by providing additional information not in the ms, or briefly explain why the item is not applicable/relevant for your study

Your answer

21-ii) Discuss if there were elements in the RCT that would be different in a routine application setting

Discuss if there were elements in the RCT that would be different in a routine application setting (e.g., prompts/reminders, more human involvement, training sessions or other co-interventions) and what impact the omission of these elements could have on use, adoption, or outcomes if the intervention is applied outside of a RCT setting.

	1	2	3	4	5	
subitem not at all important	○	○	○	○	○	essential

Does your paper address subitem 21-ii?

Copy and paste relevant sections from the manuscript (include quotes in quotation marks "like this" to indicate direct quotes from your manuscript), or elaborate on this item by providing additional information not in the ms, or briefly explain why the item is not applicable/relevant for your study

Your answer

OTHER INFORMATION

23) Registration number and name of trial registry

Does your paper address CONSORT subitem 23? *

Copy and paste relevant sections from the manuscript (include quotes in quotation marks "like this" to indicate direct quotes from your manuscript), or elaborate on this item by providing additional information not in the ms, or briefly explain why the item is not applicable/relevant for your study

"The trial was registered on ClinicalTrials.gov (NCT05368454)."

24) Where the full trial protocol can be accessed, if available

Does your paper address CONSORT subitem 24? *

Cite a Multimedia Appendix, other reference, or copy and paste relevant sections from the manuscript (include quotes in quotation marks "like this" to indicate direct quotes from your manuscript), or elaborate on this item by providing additional information not in the ms, or briefly explain why the item is not applicable/relevant for your study

This is not applicable to our study.

25) Sources of funding and other support (such as supply of drugs), role of funders

Does your paper address CONSORT subitem 25? *

Copy and paste relevant sections from the manuscript (include quotes in quotation marks "like this" to indicate direct quotes from your manuscript), or elaborate on this item by providing additional information not in the ms, or briefly explain why the item is not applicable/relevant for your study

"This study was funded by Omada Health, Inc. and Abbott Diabetes Care, Inc."

X27) Conflicts of Interest (not a CONSORT item)

X27-i) State the relation of the study team towards the system being evaluated

In addition to the usual declaration of interests (financial or otherwise), also state the relation of the study team towards the system being evaluated, i.e., state if the authors/evaluators are distinct from or identical with the developers/sponsors of the intervention.

	1	2	3	4	5	
subitem not at all important	○	○	○	○	○	essential

Does your paper address subitem X27-i?

Copy and paste relevant sections from the manuscript (include quotes in quotation marks "like this" to indicate direct quotes from your manuscript), or elaborate on this item by providing additional information not in the ms, or briefly explain why the item is not applicable/relevant for your study

Your answer

About the CONSORT EHEALTH checklist

As a result of using this checklist, did you make changes in your manuscript? *

- ○ yes, major changes
- ○ yes, minor changes
- ◉ no

What were the most important changes you made as a result of using this checklist?

Your answer

How much time did you spend on going through the checklist INCLUDING making changes in your manuscript *

We spent a several hours over multiple days reviewing the checklist and filling in the answers.

As a result of using this checklist, do you think your manuscript has improved? *

- (x) yes
- ( ) no
- ( ) Other:

Would you like to become involved in the CONSORT EHEALTH group?

This would involve for example becoming involved in participating in a workshop and writing an "Explanation and Elaboration" document

- ( ) yes
- (x) no
- ( ) Other:

Clear selection

Any other comments or questions on CONSORT EHEALTH

Your answer

STOP - Save this form as PDF before you click submit

To generate a record that you filled in this form, we recommend to generate a PDF of this page (on a Mac, simply select "print" and then select "print as PDF") before you submit it.

When you submit your (revised) paper to JMIR, please upload the PDF as supplementary file.

Don't worry if some text in the textboxes is cut off, as we still have the complete information in our database. Thank you!

Final step: Click submit !

Click submit so we have your answers in our database!

Submit Clear form

Never submit passwords through Google Forms.

This form was created outside of your domain. - Contact form owner - Terms of Service - Privacy Policy

Does this form look suspicious? Report

Google Forms
